# Recovering species demographic history from multi-model inference: the case of a Neotropical savanna tree species

**DOI:** 10.1186/s12862-014-0213-0

**Published:** 2014-10-11

**Authors:** Rosane G Collevatti, Matheus S Lima-Ribeiro, Levi Carina Terribile, Ludymila B S Guedes, Fernanda F Rosa, Mariana P C Telles

**Affiliations:** Laboratório de Genética & Biodiversidade, Instituto de Ciências Biológicas (ICB), Universidade Federal de Goiás (UFG), Cx.P. 131, Goiânia, GO 74001-970 Brazil; Laboratório de Macroecologia, Universidade Federal de Goiás (UFG), Campus Jataí, Cx.P. 03, Jataí, GO 75801-615 Brazil

**Keywords:** Coalescent simulation, Ecological niche modelling, Genetic connectivity, Habitat tracking, Multiple working hypotheses, Quaternary climate changes

## Abstract

**Background:**

Glaciations were recurrent throughout the Quaternary and potentially shaped species genetic structure worldwide by affecting population dynamics. Here, we implemented a multi-model inference approach to recover the distribution dynamics and demographic history of a Neotropical savanna tree, *Tabebuia aurea* (Bignoniaceae). Exploring different algorithms and paleoclimatic simulations, we used ecological niche modelling to generate alternative hypotheses of potential demographic changes through the last glacial cycle and estimated genetic parameters using coalescent modelling.

**Results:**

Comparing predictions from demographic hypotheses with genetic parameters of modern populations, our findings revealed a likely scenario of population decline, with spatial displacement towards Northeast Brazil from the last glacial maximum to the mid-Holocene. Subsequently, populations expanded in response to the return of the climatically suitable conditions in Central-West Brazil. Nevertheless, a wide historical refugium across Central Brazil likely maintained large populations connected throughout time. The expected genetic signatures from such predicted distribution dynamics are also corroborated by spatial genetic structure observed in modern populations.

**Conclusion:**

By exploring uncertainties inherent in multiple working hypotheses, we have shown that multi-model inference is a fruitful and efficient approach to recover the nature, timing and geographical context of the *Tabebuia aurea* population dynamic in response to the Quaternary climate changes.

**Electronic supplementary material:**

The online version of this article (doi:10.1186/s12862-014-0213-0) contains supplementary material, which is available to authorized users.

## Background

Glaciations were frequent throughout the Quaternary, with potential consequences for population genetic structure by affecting distribution and demographic dynamics of species through time [1]. Recovering population dynamics in response to past climate changes and investigating how the genetic variation was spatially structured are common and fundamental tasks in phylogeography [2,3]. However, because similar genetic patterns can arise under different demographic processes and selection, conventional methods in phylogeography based on narrative descriptions and patterns-alone interpretations (e.g. using the geographical distribution of individuals represented in an inferred gene tree) produce often dubious or indistinguishable historical demographic processes. To overcome such problem, different approaches exploring statistical and process-based modelling have been recently used for phylogeographic inference [4].

Coalescent modelling, for instance, has provided significant advances in hypothesis testing by estimating genetic parameters following predefined demographic histories (in this case, multiple working hypotheses) [5]. The hypothesis describing the most likely processes should then generate the most similar parameters for the observed pattern from real populations. This reasoning on hypothesis testing came from the multi-model inference approach, i.e. the simultaneous comparison of data from multiple hypotheses generated by different models, which is increasingly used in ecology, evolution and biogeography [6,7]. However, defining which demographic hypotheses should be simulated has been hampered by the lack of reliable and complete datasets, such as fossil records for most species worldwide see examples in [8,9]. Hence, subjective hypotheses, with little or no ecological and biogeographic realism, became common.

At the same time, ecological niche modelling (ENM, sensu Araújo & Peterson [10]) has allowed the exploration of the geographic context of species dynamics through time by hindcasting suitable climatic conditions (an n-dimensional space of climatic variables) currently occupied by species of palaeoclimatic scenarios (see limitations and assumptions of projecting ENM predictions in 11). This procedure predicts the species potential distribution over different time periods and can be further used to set demographic hypotheses [11,12]. A frequent criticism of this approach is that different ENM methods (algorithms) and palaeoclimatic simulations, which are based on different modelling assumptions or types of training data, produce different predictions [13]. Also, the difficulty of validating ENM projections (in the absence of fossil records) when their results are transferred over long time periods is another important matter of discussion [14]. Thus, ENM uncertainties would challenge objective choices, which are required for inferences of population genetics.

To overcome such problems, the full range of modelling uncertainties should be accounted for as part of the process of exploring the dynamic of species distribution through time. In this case, by exploring different assumptions about the dynamics of species ecological niche (i.e. modelling uncertainty), ENMs can actually generate multiple and independent hypotheses of species' distributional history that reflect, at the same time, ecological and biogeographical realism [11,12,15]. Subsequently, demographic hypotheses inferred from the distributional dynamic of species would be tested using coalescent analyses. Again, this idea follows the reasoning of the multi-model inference approach, which advocates that uncertainty should not be ignored, either for parameter estimation or for model setting and selection [7].

Furthermore, considering that glaciations caused unequal biotic effects worldwide, this multi-model inference approach may be especially useful to better understand the demographic dynamics of species occupying systems with complex responses to the Quaternary climate changes, such as the Neotropical savannas [16]. During the glacial periods, for instance, a drier and cooler climate occurred in South and Southeastern Brazil, and grasslands may have extended from latitudes ~ 28°/27° S to at least 20° S on savanna landscapes, although the precise age of the arid period may differ due to latitude [16]. The climate became moister and arboreal pollen dominated the savanna vegetation record only after 7,000 ^14^C yr BP, until reaching the current pattern around 4,000 ^14^C yr BP [16]. This palaeoscenario may have favoured species more adapted to drier and highly seasonal climate, consequently leading to a retraction in the geographical range of many arboreal savanna taxa that became restricted to areas with moist climatic conditions [17,18].

**Figure 1:**
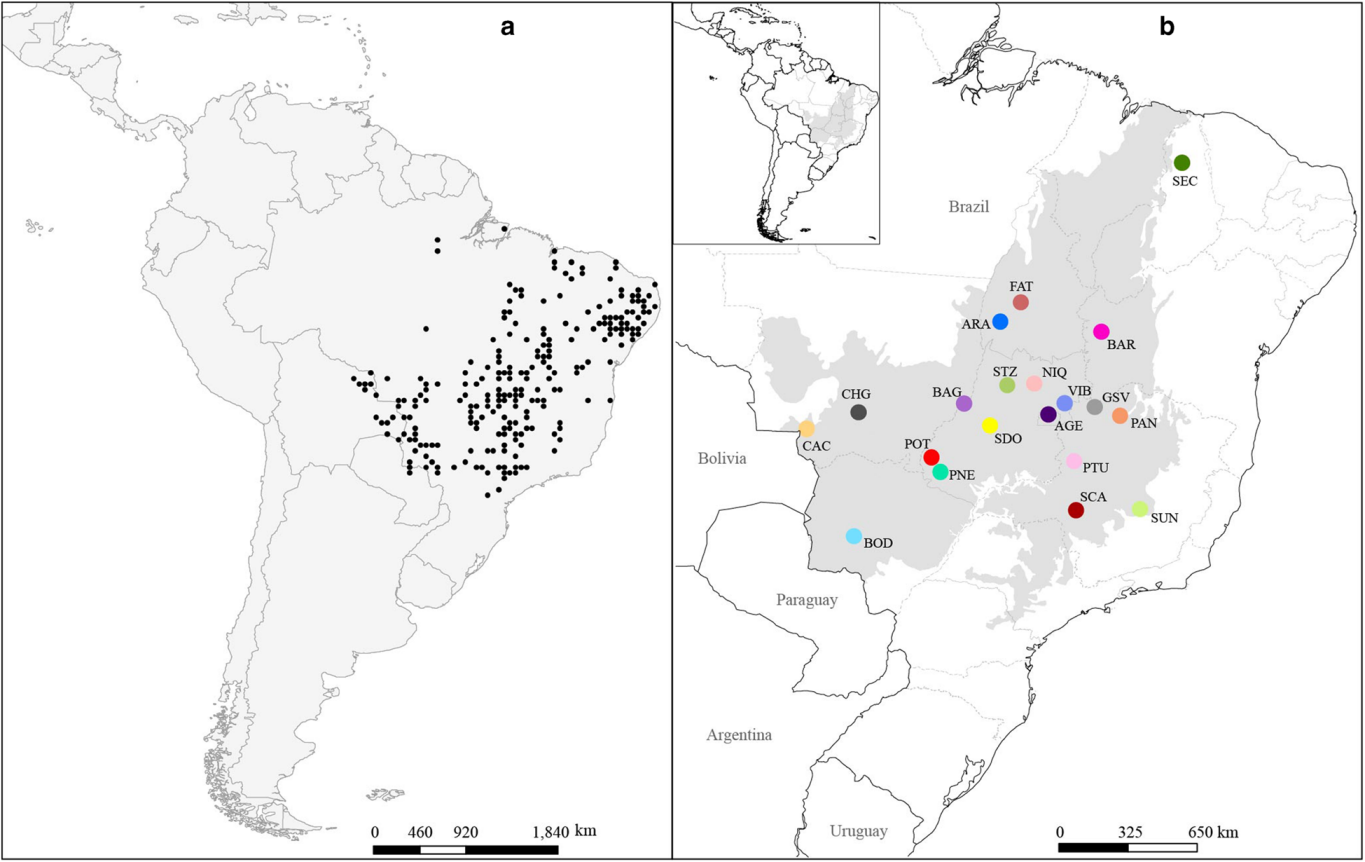
**Current geographical distribution of** ***Tabebuia aurea*** **across the Neotropics. (a)** 237 occurrence records used in ecological niche modelling; **(b)** 20 populations sampled for genetic analyses. Area in grey represents the Brazilian savannah in Central Brazil. Details on the sampled populations are provided in Additional file 1, Table S13.

Herein, we implement the multi-model inference approach to address and test multiple hypotheses concerning the effect of the late Quaternary climate oscillations on the distribution and demographic history of *Tabebuia aurea* (Silva Manso) Benth. & Hook.f. ex S.Moore (Bignoniaceae). *Tabebuia aurea* is a Neotropical tree species with a long generation time and life span, widely distributed in the seasonal savannas and wet-savanna grasslands of Central Brazil (Figure 1a), and for which there is a scarce fossil record. Moreover, general palaeovegetation reconstructions for South America constrain hypothesis testing under the coalescent theoretical background. This focal species is thus inserted into a nice context of the multi-model inference approach to elucidate its efficiency in recovering its demographic history. In particular, we explore whether the ecological and biographic hypotheses inferred from potential palaeodistribution scenarios (ENMs), along with the historical demographic dynamics simulated from coalescent analyses, reflect the observed genetic structure of modern populations.

## Results

### Ecological niche modelling

#### Species niche and consensual palaeodistribution

**Figure 2:**
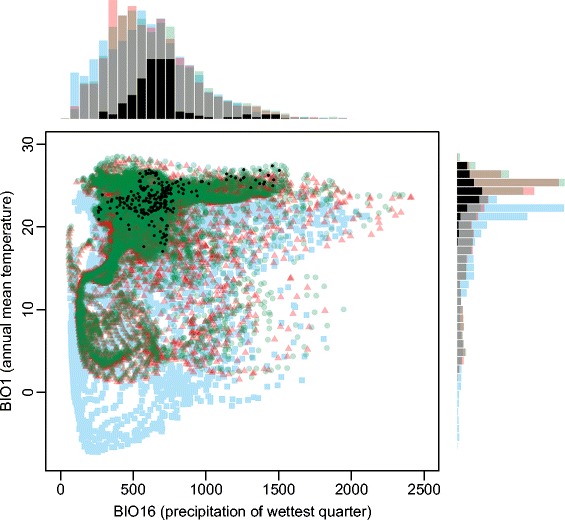
**Ecological space of climatic conditions in Neotropics during the LGM (21 ka, blue squares), mid-Holocene (6 ka, red triangles) and the present day (0 ka, green circles).** The climatic preferences from current occurrence records of *Tabebuia aurea* are represented by black dots. Note that the climatic conditions in Neotropics matching the *T. aurea's* preferences were less available during the LGM than Holocene and present-day, mainly due to temperature decrease, consequently allowing a general scenario of range expansion through the time. The bioclimatic variables were obtained from AOGCM CCSM4.

**Figure 3:**
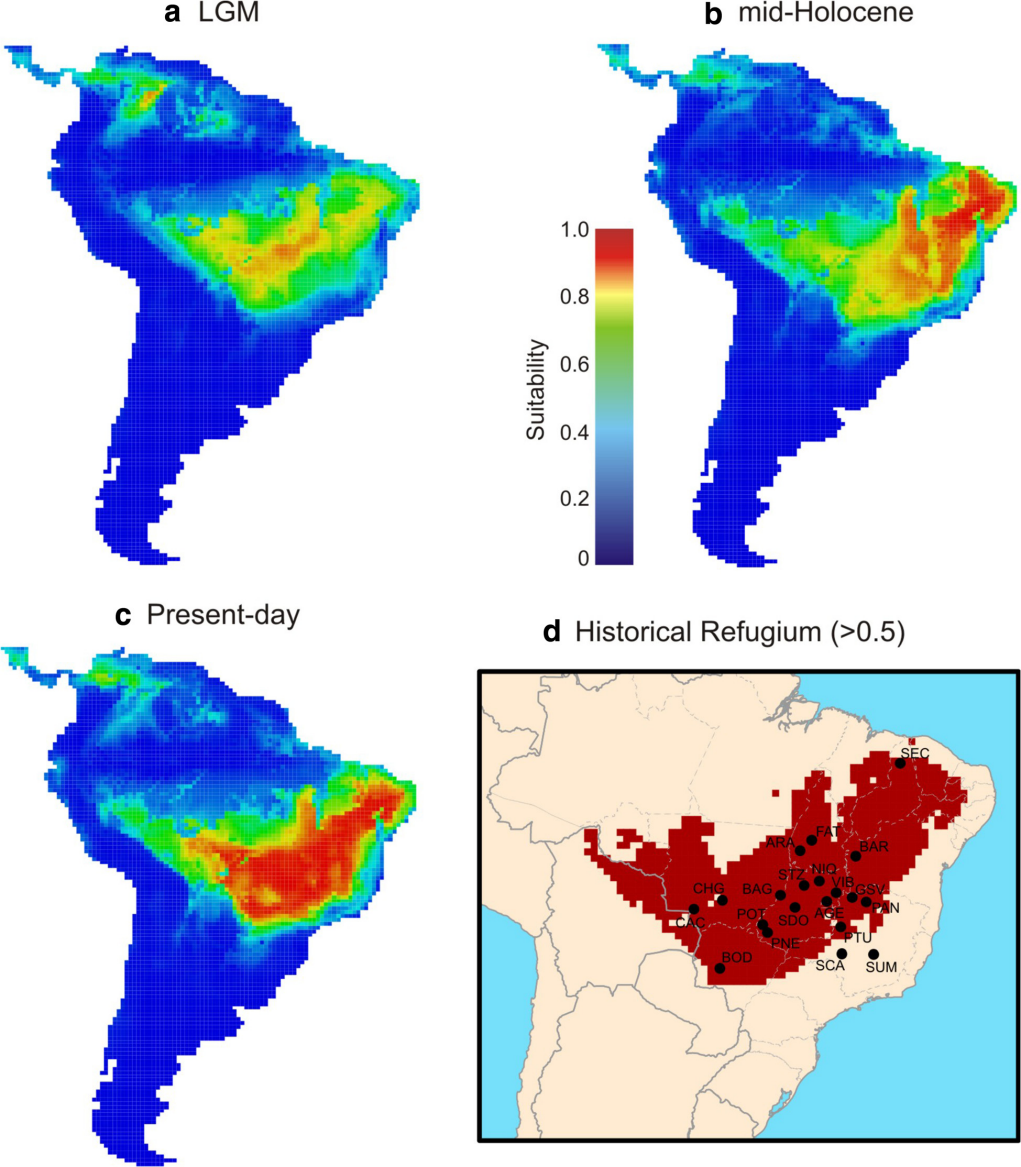
**Climatic suitability for** ***Tabebuia aurea*** **.** Maps of consensus representing the potential distribution of species across the Neotropics during the **(a)** LGM (21 ka), **(b)** mid-Holocene (6 ka), and **(c)** present day. Historical refugium **(d)** shows areas climatically suitable throughout the time.

The present-day geographic range in climatic conditions of *T. aurea* clearly shows its preference for hot and drier climates, matching the general current conditions of the Neotropical savannas (Figure 2, see also Additional file 1: Tables S1-S13). As expected, such climatic space was more restricted during the Last Glacial Maximum (LGM) than the mid-Holocene and the present day, mainly due to a temperature decrease, potentially leading to a scenario of population expansion through time in response to the increasing availability of suitable conditions across the Neotropics (see also the maps classification below). The ensembled predictions show that the potential distribution of *T. aurea* was restricted to Central Brazil during the LGM (21 ka, Figure 3a), followed by a spatial displacement towards Northeast Brazil during the mid-Holocene (6 ka, Figure 3b), and then returning to Central Brazil to the present day, expanding also towards the west of Central Brazil (Figure 3c). Despite the spatial displacements, our predictions show a wide historical refugium across Central Brazil, where higher levels of climatic suitability were maintained throughout the last glacial cycle (Figure 3d).

### Predictive uncertainties and alternative distributional hypotheses

**Figure 4:**
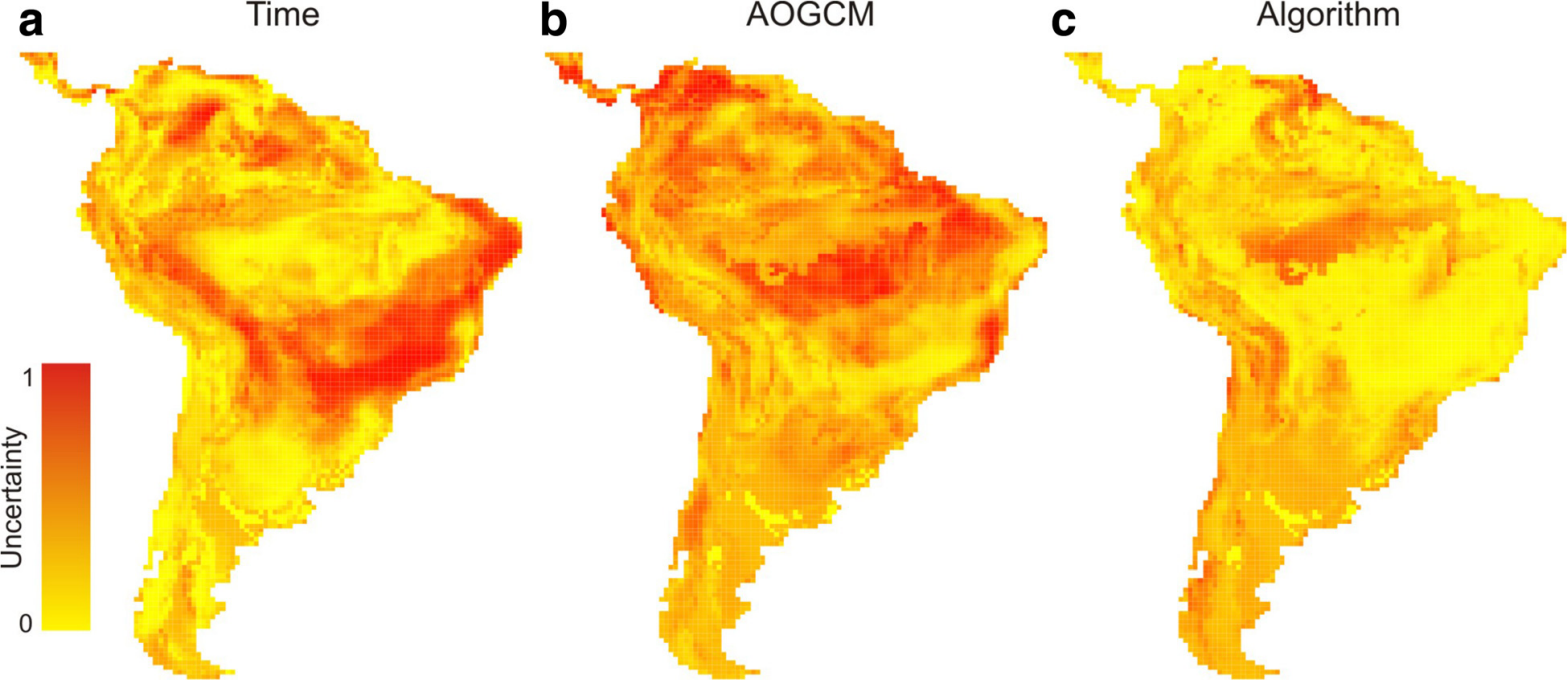
**Uncertainty from the components of ecological niche modelling. (a)** Time, **(b)** Atmosphere–ocean Global Circulation Models (AOGCMs), and **(c)** algorithms.

Considering the predictions across all 52 maps (13 algorithms *4 AOGCMs, atmosphere–ocean general circulation model) through the three time periods, the greatest variance came from AOGCMs (proportional Sum of Square = 0.35), followed by time component (SS = 0.25). Moreover, their higher uncertainties are distributed across Northeast and Central-Northeast Brazil, respectively (Figure 4a-b). The algorithms presented the lowest proportion of uncertainty (SS = 0.18), which occur outside Central Brazil, where the consensual distribution of *T. aurea* does not reach (Figure 4c, see also Additional file 2: Figures S1-S7).

In map classification, the distribution dynamics predicting range expansion through time was the most frequent scenario (Table 1 see details in Additional file 1: Tables S1-S13, and Additional file 2: Figures S1-S7). Although map classification was not dependent on the modelling components (algorithm*AOGCM) within both 6–0 ka (Pearson’s Chi-square = 104.000, *P* = 0.426; Likelihood Ratio Chi-square = 90.603, *P* = 0.783) and 21–6 ka specific periods (Pearson’s Chi-square = 104.000, *P* = 0.426; Likelihood Ratio Chi-square = 105.282, *P* = 0.392), map classification was heterogeneous between time slices (Pearson’s Chi-square = 20.744, Likelihood Ratio Chi-square = 24.552, all *P* < 0.001).

**Table 1:** **Alternative demographic hypotheses used for multi-model inference**

**Models**	***AICw***	***P***	***RS***	**ENM%**
**Retraction 21–6 ka**	-	-	1.000	1.9
**Stability 21–0 ka**	-	-	1.000	13.5
**Expansion 21–0 ka**	0.363	0.972	0.486	21.1
**Expansion 6–0 ka**	0.557	0.976	0.488	36.5
**Expansion 21–6 ka**	0.045	0.102	0.051	27.0
**Multiple Refugia 21–0 ka**	0.036	0.104	0.052	-

*AICw,* Akaike Information Criterion (AIC) weights; *P*, two-tailed probability of not rejecting the model; *RS*, relative support.

The support from palaeodistribution modelling (ENM%) is represented by the percentage of each demographic hypothesis observed through the 52 predictive maps.

### Genetic diversity and demographic parameters

Populations of *T. aurea* were genetically differentiated (*F*_*ST*_ = 0.006, SD = 0.003, *P* < 0.0001; *R*_*ST*_ = 0.209, SD = 0.023, *P* < 0.0001) with relatively high genetic diversity across its geographical range (Table 2), high levels of inbreeding within populations (*F*_*IS*_ = 0.210, SD = 0.029, *P* < 0.0001) and non-random mating among populations (*F*_*IT*_ = 0.257, SD = 0.027, *P* < 0.0001. In addition, Bayesian clustering analyses supported 19 independent genetic clusters (K = 19), also evidencing high genetic differentiation among populations (see Additional file 1: Tables S1-S13 and Additional file 2: Figures S1-S7).

**Table 2:** Genetic characterisation and demographic parameters for 20 populations of *Tabebuia aurea*

**Pop**	**N**	***A***	***Ar***	Ar*	***H***_***o***_	***H***_***e***_	***f***	***θ***	***θ - 95% interval***	***N***_***e***_	***θ - 95% interval***	***g***	***g - 95% interval***
**AGE**	30	19.2	3.6	6.3	0.860	0.930	0.075	0.023	0.010 – 0.037	0.568	0.250 – 0.934	−207.459	−476.382 - -1.057
**ARA**	12	10.4	3.5	5.7	0.710	0.898	0.209	0.025	0.006 - 0.062	0.614	0.158 – 1.544	−161.422	−436.258 - -5.331
**BAG**	26	13.4	3.4	5.6	0.727	0.885	0.178	0.021	0.009 - 0.054	0.515	0.213 – 1.338	−170.238	−473.217 - -43.702
**BAR**	15	12.2	3.5	5.9	0.710	0.906	0.217	0.030	0.007 - 0.079	0.740	0.186 – 1.972	−270.417	−498.155 - -33.706
**BOD**	22	16.1	3.6	6.3	0.748	0.928	0.194	0.039	0.008 - 0.109	0.972	0.205 – 2.733	−148.207	−415.330 - -11.316
**CAC**	30	16.7	3.6	6.3	0.715	0.929	0.230	0.025	0.009 - 0.074	0.617	0.236 – 1.848	−263.149	−491.464 - -45.690
**CHG**	24	13.9	3.4	5.7	0.680	0.884	0.231	0.022	0.006 - 0.050	0.555	0.149 – 1.260	−197.548	−471.766 - -18.742
**FAT**	35	17.0	3.6	6.1	0.687	0.917	0.251	0.022	0.007 - 0.045	0.541	0.170 – 1.122	−163.550	−467.321 - -46.328
**GSV**	8	7.5	3.3	5.3	0.780	0.874	0.107	0.040	0.005 - 0.116	1.001	0.113 – 2.893	−220.179	−468.852 - -41.132
**NIQ**	28	13.2	3.4	5.6	0.675	0.883	0.236	0.016	0.009 - 0.024	0.405	0.219 – 0.610	−253.793	−486.822 - -1.000
**PAN**	23	15.2	3.6	6.2	0.669	0.926	0.278	0.036	0.008 - 0.129	0.902	0.210 – 3.218	−157.302	−473.707 - -68.541
**PNE**	16	14.5	3.6	6.2	0.677	0.929	0.272	0.039	0.008 - 0.101	0.979	0.202 – 2.523	−221.599	−482.206 - -4.158
**POT**	16	12.1	3.3	5.5	0.744	0.875	0.150	0.021	0.004 - 0.060	0.525	0.097 – 1.500	−202.442	−469.204 - -9.047
**PTU**	32	15.9	3.4	5.8	0.669	0.896	0.253	0.022	0.012 - 0.036	0.540	0.295 – 0.901	−285.170	−485.482 - -23.824
**SCA**	29	9.7	3.1	4.7	0.664	0.814	0.185	0.013	0.006 - 0.022	0.323	0.140 – 0.551	−206.995	−489.117 - -1.396
**SDO**	3	3.9	3.2	-	0.606	0.932	0.350	-	-	-	-	-	-
**SEC**	4	4.5	3.1	-	0.742	0.852	0.129	-	-	-	-	-	-
**STZ**	17	11.2	3.4	5.5	0.749	0.885	0.153	0.029	0.007 - 0.095	0.733	0.173 – 2.378	−306.082	−483.282 - -1.000
**SUM**	14	9.0	3.2	4.9	0.636	0.845	0.247	0.017	0.009 - 0.033	0.437	0.229 – 0.821	−272.212	−489.161 - -19.884
**VIB**	30	12.3	3.3	5.3	0.660	0.865	0.237	0.015	0.009 - 0.024	0.384	0.216 – 0.600	−173.979	−483.035 - -15.963
**Mean**		36.2	3.7	6.7	0.705	0.893	0.209	-	-			-	-
**SD**		9.3	0.1	0.3	0.057	0.033	0.068	-	-			-	-
**Overall**	414							7.033	0.858 - 1.000	2,109	1,372 – 38,400	−0.077	−1.706 - 1.244

N – number of individuals sampled; *A* – mean number of alleles per locus; *Ar* – allelic richness; Ar* - allelic richness excluding populations SEC and SDO; *H*_*o*_ - observed heterozygosity; *H*_*e*_ – expected heterozygosity; *f* – inbreeding coefficient (all values are significant P < 0.01); *θ* - coalescent parameter; 95% credibility interval around the estimate; *N*_*e*_ – effective population size; *g* - exponential growth parameter (all values are significantly different from zero based on the credibility interval around the estimates).

Coalescent analyses supported a low mutation parameter (*θ*) and small effective population sizes for all populations (Table 2). Using the mutation parameter over all populations (*θ* = 7.033), TMRCA dated 8.439 ka (95% CI = 5.489 – 153.600 ka) and effective population size was *N*_*e*_ = 2,109 (95% CI = 1,372 – 38,400). Gene flow was high among all population pairs (*N*_*e*_*m* ≥ 1.00, see Additional file 2: Figures S1-S7).

### Model selection

**Figure 5:**
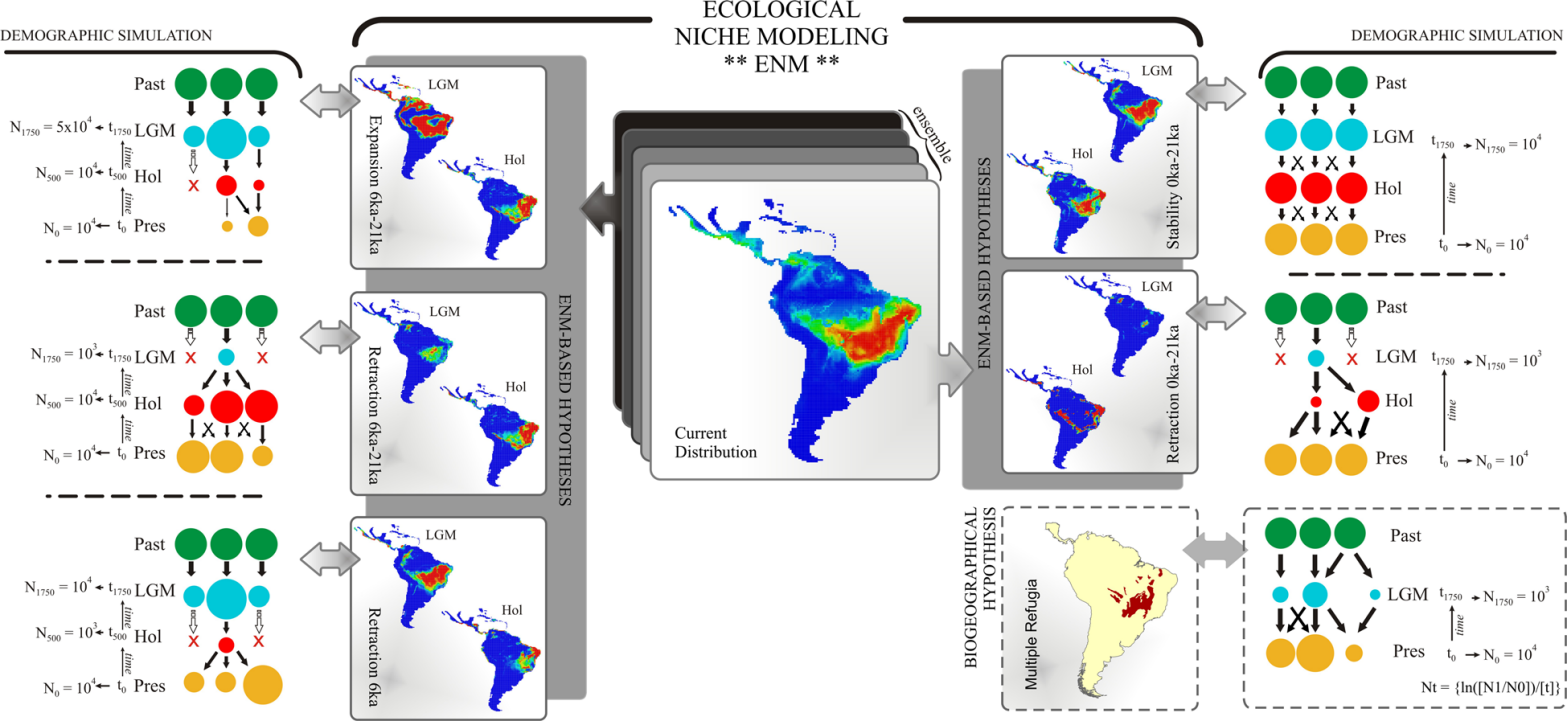
**Demographic history scenarios simulated for** ***Tabebuia aurea*** **and their palaeodistributional representations.** Circles represent hypothetical demes and indicate population stability or shrinkage through the time. LGM, last glacial maximum; Hol, mid-Holocene; Pres, present day; N0, N500 and N1750, effective population size at time t0 (present day), time t500 (500 generations ago, matching mid-Holocene), and time t1750 (1750 generations ago, matching LGM), respectively; Nt, logarithm function for effective population size variation in coalescent simulation. The migration rate was 0.01/generation.

Similar to the ENMs predictions, the two models of population expansion through time (Expansion 6–0 ka and Expansion 21–0 ka, see Figure 5 for details on demographic models) were the best-supported hypotheses for predicting the observed genetic parameters for *T. aurea,* using either two-tailed probability (*P*) or *AICw* (Akaike Information Criteria weights) criteria for model selection (Table 1). Hypotheses predicting population retraction or stability retrieved 100% of the values (>0.988) above the observed mean genetic diversity for the 20 populations of *T. aurea*. Thus, relative support (*RS*) was 1.000 and *AICw* and *P* could not be estimated. In addition, the hypotheses "Expansion 21–6 ka" and "Multiple Refugia 21–0 ka" retrieved a greater proportion of genetic diversity below the observed values (see Additional file 2: Figures S1-S7).

### Spatial patterns in genetic diversity

We found no significant relationship between the geographical distance and the genetic differentiation among pairs of populations (Mantel test, *r2* = 0.035, *P* = 0.144). However, quantile regressions showed clear effects of climate change on the genetic structure of *T. aurea*. Relationships resembling triangular-shaped envelopes show that populations in less climatically suitable areas during the LGM and present day presented high or low genetic diversity (*H*_*e*_) and allelic richness (Ar*), whereas populations in more suitable areas had only high *H*_*e*_ and Ar*. An inverse pattern occurred at 6 ka (see Additional file 1: Tables S1-S13.

Likewise, lower values of both *H*_*e*_ and Ar* are associated with populations in areas where climatic suitability increased from the LGM to the mid-Holocene, whereas higher values occurred in populations located in regions where suitability increased from the mid-Holocene to the present (see Additional file 1: Tables S1-S13). Notwithstanding, distances from the centroids often showed weak or no significant relationships with either *H*_*e*_ or Ar* (see Additional file 1: Tables S1-S13).

## Discussion

### Inferences on demographic history

Our findings showed clear evidence that the current pattern in *T. aurea* genetic diversity is the result of population expansion through time. The general scenarios predicting "Expansion" were the most frequent among ENM predictions and the best-supported models among demographic simulations. Moreover, genetic analyses also showed that all populations present a negative growth parameter (although the credibility intervals were large) and small effective population sizes, which in turn may also be the outcome of smaller populations in the past than in the present.

In fact, a slow and long population expansion lasting 1750 generations from the LGM to the present ("Expansion 21–0 ka") or a fast and brief expansion from the mid-Holocene (6 ka) to present ("Expansion 6–0 ka") retrieved similar results of genetic diversity and were equally supported in our analyses. However, some theoretical expectations favour the scenario of "Expansion 6–0 ka". For example, the probability of coalescence is inversely related to the number of gene copies [19], therefore populations that experienced population retraction followed by expansion may present the most coalescence just before the demographic expansion when population size was small (see [20] for a review). Thus, because the TMRCA for *T. aurea* lineages dated from the early- to mid-Holocene (~8 ka), our results are more congruent with the hypothesis "Expansion 6–0 ka". We believe therefore that this scenario of a fast and brief population expansion from the mid-Holocene (6 ka) to the present should be considered for *T. aurea* hereafter.

### Geographical context: inferences on genetic and spatial connectivity

Along with population expansion from the mid-Holocene, the spatial displacements in climatically suitable areas through time also affected the current genetic pattern in *T. aurea*. Populations with lower values of both genetic diversity and allelic richness are located in the Northeast edge of the *T. aurea* geographical range, a region potentially occupied only during the mid-Holocene. Following the abundant-centre model (i.e. loss of genetic variability due to genetic drift and inbreeding in peripheral populations with low effective population sizes, [21]), we hypothesize that populations of *T. aurea* at the northeast edge already had depleted genetic diversity due to small effective sizes, which may have in turn reduced individual fitness and population adaptability, and constrained the scale up in genetic diversity. Moreover, the northeastward range shift may have led to the colonisation of new sites with low genetic diversity and allelic richness due to allele surfing (see [22]).

In addition, evidence that most populations are inside the historical refugium suggests that even under demographic retraction or spatial displacements, a wide climatically stable area available throughout the last glacial cycle favoured population persistence and genetic connectivity across Central Brazil, hence the high genetic diversity and polymorphism observed in modern populations. Interestingly, the two populations outside the historical refugium, SCA and SUM, which were potentially disconnected from the others in some periods, presented the lowest values of both genetic diversity (*H*_*e*_) and allelic richness (Ar*). Moreover, the evidence of historical connectivity among populations of *T. aurea* also reinforces the observed pattern of high gene flow (*N*_*e*_*m* ≥ 1.00) and low genetic differentiation (*F*_*ST*_). The genetic connectivity is also in agreement with patterns of pollen dispersal observed elsewhere. *Tabebuia aurea* has a mixed mating system, with potential long distance pollen (greater than 2.5 km) and seed dispersal [23]. However, high proportions of self-pollination and biparental inbreeding have been observed, which may have led to high inbreeding within population and a high fixation index (*F*_*IS*_).

### Predictive uncertainties: are the hypotheses and inferences reliable?

The spatial pattern of predictive uncertainties suggests that algorithms converge in their main prediction about the distribution of the species across Central Brazil through time. Moreover, the high levels of variance of the time component indicate that ENMs were able to get the effects of climate changes from the *T. aurea* distributional dynamics throughout the last glacial cycle, despite some methodological noise.

The set of 52 maps from ecological niche modelling thus reflects a full range of potential climate change effects on the distributional dynamics of *T. aurea* across Neotropics, supporting alternative demographic hypotheses that are ecologically and biogeographically valid. Along with ENMs, the validity of such hypotheses is still evident by the fact that the expected demographic histories realistically predicted the current genetic structure of *T. aurea* when simulated using coalescent models. Therefore, we consider both hypotheses and inferences built by coupling niche modelling and coalescent simulations to be reliable as approached here. Most importantly, our framework provides explicit mechanisms to include and analyse a complete range of uncertainties as required for hypothesis testing in any multi-model inference approach.

## Conclusions

Exploring uncertainties inherent to the different modelling techniques, we recovered the demographic history of *T. aurea* and showed that the pattern of genetic diversity and allelic richness may be the outcome of range expansion from the LGM to the present. Besides range expansion, a wide suitable region throughout the last glacial cycle was evidenced in Central Brazil. This wide historical refugium may have buffered the deleterious effect of demographical retraction on genetic diversity due to the long-term population persistence and connectivity, maintaining a relatively high level of genetic diversity and allelic richness in most populations with significant but low genetic differentiation among them.

Although we could not validate the palaeodistribution hypotheses due to the lack of fossil records (see [14], [9]), we believe that our biogeographic hypothesis is reliable because it matches the general pattern of fossil records for Central Brazil [16]. Also, the demographic hypotheses tested here reflect a complete range of potential climatic effects on species distributional dynamic through the time. Moreover, the ensemble among predictions also reflects a likely spatial context of the demographic dynamic with explicit mechanisms to analyse uncertainties and hypothesis reliability, although with some methodological noise (i.e. predictive uncertainties from algorithms and AOGCMs). Thus, we believe that the multi-model inference approach used in this study is a useful framework to recover the nature, timing and geographical context of population dynamics in response to Quaternary climate changes, as well as to better understand the mechanisms involved in the origin and maintenance of species genetic diversity.

## Methods

### Setting-up working hypotheses

#### Hindcasting species distribution

To explore uncertainties across the ecological niche modelling and generate alternative demographic hypotheses, we considered palaeoclimatic simulations from four coupled atmosphere–ocean general circulation models (see details on Additional file 2: Figures S1-S7) AOGCM (CCSM4, CNRM-CM5, MIROC-ESM and MRI-CGCM3) available at CMIP5 (http://cmip-pcmdi.llnl.gov/cmip5/) and PMIP3 databases (https://pmip3.lsce.ipsl.fr/). Climatic layers were obtained for pre-industrial (representing current climate conditions), mid-Holocene (6 ka) and Last Glacial Maximum (LGM; 21 ka) to characterize climate oscillations, hence the palaeodistribution dynamics through the last glacial cycle.

We used monthly simulations of precipitation, and mean, maximum and minimum temperatures downscaled to a grid of cells with 0.5° spatial resolution encompassing the Neotropical region (see [24] for details), to compute the 19 bioclimatic variables defined in WorldClim (www.worldclim.org). Precipitation and temperature are the basic components of climatic axis of species niche and are frequently used as surrogates of water and energy availability, which are considered the main drivers of species distributions at macroscales [25]. Five bioclimatic variables were selected from the 19 - annual mean temperature, annual temperature range, precipitation of the wettest month, precipitation of the driest month, and precipitation of the warmest quarter - using a factor analysis based on the correlation matrix to minimise collinearity problems when building the ENMs (the variables with the highest loadings in the first five Varimax rotated eigenvectors; see Additional file 2: Figures S1-S7). Along with these variables, in all AOGCMs we included subsoil pH (30–100 cm; from the Harmonized World Soil Database – ver. 1.1, FAO/ IIASA/ISRIC/ISS-CAS/JRC 2009) as a “constraint variable” to improve the ENM predictions (i.e., because these soil data are not available for the past and future, we are assuming that their change at broad geographic scales is much smaller than changes in climatic variables – but even so they constrain the distribution of species at any time). Indeed, the inclusion of this soil variable was previously demonstrated to improve the predictive performance of ENMs for tree species in the Brazilian Cerrado [12].

Contemporary occurrence records of *T. aurea* (Figure 1a, Additional file 2: Figures S1-S7) were gathered across the Neotropics from the online databases GBIF (Global Biodiversity Information Facility http://www.gbif.org/) and Species Link (http://splink.cria.org.br/). The climatic conditions occupied by *T. aurea* along the 237 georeferenced occurrences were then fitted with the climatic space (i.e. the species niche) and used to identify potential climatically suitable areas for species distribution in geographical space. By hindcasting this model onto the mid-Holocene and LGM climatic scenarios [26], we tracked the species distribution dynamics though time.

Current and past potential distributions were obtained using 13 algorithms based on different assumptions to estimate species niche (Additional file 2: Figures S1-S7). Species presence and pseudo-absences (randomly selected on background region with the same proportion of presence records) were randomly divided into 75% for calibration and 25% for evaluation, and this process was repeated 50 times. ENM algorithms were implemented in the computational platform Bioensembles (see [27]) which is based on the ensemble approach [13]. Bioensembles directly executes some distance-based methods (e.g., Euclidian and Mahalanobis distance) and Bioclim, and it also performs algorithms from other sources (e.g., GARP from OpenModeller, http://openmodeller.sourceforge.net/), along with the integration with external software (e.g. MAXENT is executed from the original software) and methods implemented in R (e.g. GBM, FDA, GLM, GAM).

The combinations of all modelling components (13 algorithms *4 AOGCMs) resulted in 52 consensual predictive maps for each time period. The consensual maps were generated by computing the mean suitability across all 52 models weighted by predictive performance according to the True Skill Statistics (TSS) of each model (Additional file 2: Figures S1-S7). These maps reflect the resulting range of potential climate change effects on the distribution dynamics of focal species, and will help to calibrate coalescent models (see below). Also, consensual maps were used to identify historical refugia for *T. aurea*. In this case, a cell was considered climatically stable if the species was predicted to be present (considering a threshold in suitability values of 0.3) in that cell during the three time periods (the LGM, the mid-Holocene and the present).

Finally, we applied a hierarchical ANOVA using the predicted suitability from all models (13 ENMs *4 AOGCMs *3 Time Periods) as a response variable to disentangle and map the uncertainties in the potential distribution due to modelling components (i.e. ENMs, AOGCMs) and the effects of climate change on the species distribution through the time. The ANOVA was hierarchically designed to maintain ENM and AOGCM components nested into the time component, but crossed by a two-way factorial design within each time period (see 25 for details about hierarchical design).

### Inferring demographic hypotheses

We classified the species distribution dynamics predicted from all 52 predictive maps according to three general scenarios: i) "Range Stability": no difference in range size through time; ii) "Range Retraction": range size decreases from one time period to another; and iii) "Range Expansion": range size increases from one time period to another. Classifications were first made by visual inspection and then by computing the difference in range size between combinations of time slices: 21 – 6 ka, 6 ka – present, and 21 ka - present), but keeping constant the combination of algorithm and AOGCM in the three time periods (see Additional file 2: Figures S1-S7, and Additional file 1: Tables S1-S13). The cut-off for the difference in number of grid cells for each category was from −199 to 199 for "Range Stability", difference > 199 for "Range Retraction", and difference < −199 for “Range Expansion". We used a log-linear analysis to verify the relationship between map classification and the sources of variation among them; i.e. algorithms and AOGCMs. As the result did not converge, probably due to the low degrees of freedom, we performed an association test among them ((Map)*(Classification 21–6 ka)*(Classification 6–0 ka)).

The comparison of maps among time slices resulted in five alternative demographic hypotheses (see details in Additional file 2: Figures S1-S7): 1) Range Expansion between 21 and 0 ka; 2) Range Expansion between 21 and 6 ka; 3) Range Expansion between 6 and 0 ka; 4) Range Retraction between 21 and 6 ka; and 5) Range Stability. Along with these five hypotheses supported by ENMs, we established a sixth hypothesis, "Multiple Refugia", derived from general palaeovegetational reconstructions for late Quaternary in South America [28] and supported in previous phylogeographic studies in Neotropics [9,18]. The resulting six hypotheses (Table 1) thus offer a complete set of alternative population dynamics to calibrate coalescent models (Figure 5).

### Demographic history simulations

The genetic signatures from the six alternative demographic hypotheses were simulated using coalescent analysis implemented in the software ByeSSC [29]. To allow direct comparison between predicted and observed genetic structures and to determine the best-supported hypothesis, we used demographic parameters observed for focal species to calibrate the demographic models. For instance, we considered 11 microsatellite loci and 20 demes coalescing in the past under a mutation rate and generation time matching estimates for *T. aurea's* populations (see details in the next section). Because coalescent theory is set backward through time, population dynamics were simulated from t0 (present) to t500 and t1750 generations ago (at 6 ka and 21 ka, respectively). For all simulations, populations started invariably with N0 = 10,000 individuals (again, the effective population size estimated for modern populations) and grew according the theoretical expectation from each demographic hypothesis (see details in Figure 5). Due to this backward view, negative growth across coalescent simulations implies population expansion from the past to the present, whereas a positive growth implies populations smaller now than in the past.

The demographic hypothesis predicted by "Retraction 21–6 ka" was then simulated by keeping the population size constant from the present until 6 ka (i.e. N0 = N500 = 10,000) and then applying an exponentially positive population growth from 6 ka to 21 ka to achieve N1750 equal to 50,000 (i.e. in a forward view, the population reduced from 21 ka to 6 ka, keeping constant and small until the present) (Figure 2). In contrast, the "Expansion 21–0 ka" hypothesis was simulated applying exponentially negative population growth from the present to 21 ka until attaining N1750 equal to 1,000. This simulation differed from that “Expansion 21–6 ka" hypothesis because in the latter we kept a constant population size from 0 ka to 6 ka and then the population size was reduced until 21 ka, with N1750 equal to 1,000. For the "Expansion 6–0 ka" hypothesis, population size was reduced until 6 ka, with N500 = 1,000 and then the population exponentially grew until 21 ka, attaining the same size at N0 (N1750 = 10,000). Migration was simulated considering all current deme descendants from lineages originally in deme 1 at t generations ago, meaning that as the coalescent tree builds back through time, there is a 0.01/generation chance that each lineage in deme *x* will migrate to deme 1 every time. For the "Retraction" hypothesis, we considered that each lineage in deme *x* will migrate to deme 1 and then shrink until extinction at *t* generations ago. Finally, the "Multiple Refugia" hypothesis was simulated from a finite island model [30]; i.e. current populations are descendant from lineages originally in the demes at t1750 generations ago, meaning that as the tree builds back through time, population shrink with N1750 equal to 1,000 and a chance of 0.01/generation for changing migrants among demes every time.

### Model parameterisation: genetic diversity, demographic parameters and population structure for focal species

We estimated the genetic structure of modern populations of *T. aurea* to calibrate coalescent models and allow further comparisons with predictions from alternative demographic hypotheses. For this, 414 individuals in 20 populations throughout the Brazilian savanna were sampled (Figure 1b, Additional file 2: Figures S1-S7) and genotyped for 11 nuclear microsatellite loci (see Additional file 3). We focused our sampling efforts on the savannas of Central-West Brazil due to the current higher abundance of *T. aurea*. Sampling was not performed in conservation units and thus did not require any license. Vouchers were compared to herbarium material from the Federal University of Goiás (Universidade Federal de Goiás) in Goiânia.

The parameters for demographic simulations were obtained using coalescent analyses implemented in the software Lamarc 2.1.8 [31]. The demographic parameter theta, *θ* = 4*μN*_*e*_ (coalescent or mutation parameter for a diploid genome, where *N*_*e*_ is the effective population size), was estimated using a Markov Chain Monte Carlo (MCMC) approach [32]. We also explored changes in effective population size by estimating the demographic parameter *g* (exponential growth rate), where *θ*_*t*_ = *θ*_*now*_ exp(−*gt*), and *t* is the time to coalescence in mutational units [33]. To access historical genetic connectivity, we estimated the number of migrants per generation from scaled migration rate, M = 4*N*_*e*_*m*/*θ*, where *m* is the migration rate. Because of the low sample size, demographic parameters were not estimated for populations SDO and SEC (see Figure 1b).

To characterize the population structure and determine the number of demes in simulations, we first verified if populations were genetically differentiated. We obtained Wright’s F-statistics, *F*_*IT*_*, F*_*ST*_*,* and *F*_*IS*_ [34] from the analysis of variance of allele frequencies, implemented in the software FSTAT 2.9.3.2 [35]. We also estimated the Slatkin’s *R*_*ST*_ [36] based on the variance in allele sizes to verify the contribution of stepwise-like mutations to genetic diversity and test the hypothesis that *F*_*ST*_ = *R*_*ST*_ using the software SPAGeDi 1.4 [37]. Next, we performed a Bayesian clustering simulation to access the number of discrete genetic clusters (K) using the software STRUCTURE 2.3.4 [38].

Time to most recent common ancestor (**TMRCA**) and effective population size were estimated from the mutation parameter *θ* ([39], see also [39]) using a generation time of 12 years (based on flowering time; RG Collevatti, unpublished data). Based on the comparison of *F*_*ST*_ and *R*_*ST*_ (see results), we used the highest mutation rate reported for microsatellite markers in plants; i.e. 1.0 × 10^−2^ mutation per allele per generation (95% CI = 0.89 × 10^−2^ to 1.2 × 10^−2^) [40].

Finally, genetic diversity parameters such as number of alleles per *locus* (*A*), allelic richness (*Ar*) for reference sample size of two individuals, expected heterozygosity (*H*_*e*_) under Hardy-Weinberg equilibrium and the inbreeding coefficient (*f*) were estimated using the software FSTAT 2.9.3.2 [35] to further comparisons with predicted genetic signatures from alternative demographic hypotheses.

### Data analyses

#### Model selection

The genetic diversity predicted across 2,000 simulations was compared with the empirical genetic diversity (mean expected heterozygosity under Hardy-Weinberg expectations for the 20 populations) to determine which demographic dynamic was best supported. Two-tailed probability (*P*) and Akaike Information Criterion (AIC) were estimated for each demographic model. We also calculated the relative support (*RS*) given by the proportion of simulated values higher than observed for each model. The log-likelihood was estimated as the product of the height of the empirical frequency distribution at the observed value of diversity by the maximum height of the distribution (see BayeSSC website, www.stanford.edu/group/hadlylab/ssc/index.html). AIC was transformed into AIC weighting, given by exp [−0.5(AIC – AICmin)] [41].

### Spatial pattern in genetic diversity

We used spatially explicit analysis to detect gradients in observed genetic diversity or sectors of low genetic diversity in response to late Quaternary climate oscillations. To test for spatial structures, the linearized *F*_*ST*_ and geographical distances (in logarithm scale) among pairs of sampled populations were correlated using a Mantel test. Statistical significance was established from 10,000 random permutations using the software Arlequin 3.11 [42].

We also analysed the relationship of climatic suitability and stability through time with genetic diversity (*H*_*e*_) and allelic richness (Ar*) using quantile regression [43]. For this, we calculated the difference of ensembled suitabilities among time intervals (i.e. 21 – 6 ka and 6 – 0 ka) as a measure of climate stability through time. Next, we analysed whether historical changes in species’ geographical range generated a cline spatial pattern in genetic diversity and allelic richness due to expansion, contraction and spatial displacements of climatically suitable conditions. For this, we obtained, for each analysed population, the distance from the centroids of the potential distributions at present, 6 ka and 21 ka, as well as to the centroid of historical refugium. Then, we performed quantile regressions of both genetic parameters (*H*_*e*_ and Ar*) against these spatial distances.

## Availability of supporting data

Additional accessibility data is provided as Additional file.

## Additional files

Additional file 1: Tables S1-S13. Additional data on ecological niche modeling, population structure analyses and coalescent analyses. Additional file 2: Figures S1-S7. Additional data o ecological niche modelling, population structure analyses and spatial pattern in genetic diversity. Additional file 3:
**Details on genotyping procedure.**
