# Supplementary material for: Recovering species demographic history from multi-model inference: the case of a Neotropical savanna tree species
Source: BMC Evol Biol. 2014 Oct 11;14:213. doi: 10.1186/s12862-014-0213-0 (PMC4205293; doi:10.1186/s12862-014-0213-0)
Supplement: Additional file 1: Tables S1-S13. — Additional data on ecological niche modeling, population structure analyses and coalescent analyses. [file 12862_2014_213_MOESM1_ESM.doc]

**Additional file 1**

**Table S1.** Uncertainty of the modelling components from ecological niche modelling predictions for *Tabebuia aurea* as revealed by hierarchical ANOVA. SS: sum of square.

| **Source of variation** | **Median SS** | **Minimun** | **Maximum** |
| --- | --- | --- | --- |
| **TIME** | 0.25 | 0.00 | 0.94 |
| **AOGCM** | 0.35 | 0.02 | 0.96 |
| **ENM** | 0.18 | 0.004 | 0.81 |
| **Residual** | 0.09 | 0.003 | 0.40 |

**Table S2.** Classification of the 52 predictive maps following the three general scenarios of distribution dynamic through the time: range stability, retraction and expansion.

| **AOGCM** | **ENM** | **Range Size** | | | **Range Shift** | | | | | |
| --- | --- | --- | --- | --- | --- | --- | --- | --- | --- | --- |
|  |  |  |  |  | **21-6 ka** | | **6-0 ka** | | **21-0k** | |
|  |  | **21 ka** | **6 ka** | **0 ka** | **shift** | ***H*** | **shift** | ***H*** | **shift** | ***H*** |
| CCSM | BioClim | 1357 | 873 | 1629 | -484 | r | 756 | e | 272 | e |
| CCSM | ENFA | 2321 | 2498 | 2609 | 177 | e | 111 | e | 288 | e |
| CCSM | EuclidDist | 1419 | 1715 | 1840 | 296 | e | 125 | e | 421 | e |
| CCSM | FDA | 1376 | 1420 | 1550 | 44 | s | 130 | e | 174 | e |
| CCSM | GAM | 1575 | 1278 | 1578 | -297 | r | 300 | e | 3 | s |
| CCSM | GBM | 1249 | 1221 | 1477 | -28 | s | 256 | e | 228 | e |
| CCSM | GLM | 1597 | 1222 | 1688 | -375 | r | 466 | e | 91 | s |
| CCSM | GowerDist | 1485 | 1860 | 1931 | 375 | e | 71 | s | 446 | e |
| CCSM | MahalDist | 1978 | 2046 | 2151 | 68 | s | 105 | s | 173 | e |
| CCSM | MARS | 1349 | 1401 | 1477 | 52 | s | 76 | s | 128 | e |
| CCSM | MaxEnt | 1471 | 901 | 1527 | -570 | r | 626 | e | 56 | S |
| CCSM | NNet | 1411 | 1038 | 1700 | -373 | r | 662 | e | 289 | E |
| CCSM | RndFor | 1278 | 1505 | 1536 | 227 | e | 31 | s | 258 | E |
| CNRM | BioClim | 1117 | 854 | 1601 | -263 | r | 747 | e | 484 | E |
| CNRM | ENFA | 2601 | 2262 | 2599 | -339 | r | 337 | e | -2 | S |
| CNRM | EuclidDist | 1912 | 1531 | 1809 | -381 | r | 278 | e | -103 | S |
| CNRM | FDA | 1620 | 1365 | 1507 | -255 | r | 142 | e | -113 | R |
| CNRM | GAM | 1700 | 1070 | 1594 | -630 | r | 524 | e | -106 | S |
| CNRM | GBM | 1325 | 1081 | 1435 | -244 | r | 354 | e | 110 | S |
| CNRM | GLM | 1464 | 945 | 1573 | -519 | r | 628 | e | 109 | S |
| CNRM | GowerDist | 1912 | 1436 | 1764 | -476 | r | 328 | e | -148 | R |
| CNRM | MahalDist | 1780 | 1370 | 1719 | -410 | r | 349 | e | -61 | S |
| CNRM | MARS | 1569 | 1293 | 1489 | -276 | r | 196 | e | -80 | S |
| CNRM | MaxEnt | 1387 | 868 | 1482 | -519 | r | 614 | e | 95 | S |
| CNRM | NNet | 1213 | 1374 | 1477 | 161 | e | 103 | s | 264 | E |
| CNRM | RndFor | 1504 | 1295 | 1589 | -209 | r | 294 | e | 85 | S |
| MIROC | BioClim | 1642 | 1759 | 1855 | 117 | e | 96 | s | 213 | E |
| MIROC | ENFA | 2527 | 2302 | 2599 | -225 | r | 297 | e | 72 | S |
| MIROC | EuclidDist | 1937 | 1895 | 2098 | -42 | s | 203 | e | 161 | E |
| MIROC | FDA | 1563 | 1808 | 1934 | 245 | e | 126 | e | 371 | E |
| MIROC | GAM | 1716 | 1676 | 1749 | -40 | s | 73 | s | 33 | S |
| MIROC | GBM | 1310 | 1590 | 1652 | 280 | e | 62 | s | 342 | E |
| MIROC | GLM | 1867 | 1724 | 1764 | -143 | r | 40 | s | -103 | S |
| MIROC | GowerDist | 2151 | 2232 | 2421 | 81 | s | 189 | e | 270 | E |
| MIROC | MahalDist | 2156 | 2220 | 2492 | 64 | s | 272 | e | 336 | E |
| MIROC | MARS | 1372 | 1555 | 1733 | 183 | e | 178 | e | 361 | E |
| MIROC | MaxEnt | 1290 | 1562 | 1614 | 272 | e | 52 | s | 324 | E |
| MIROC | NNet | 1522 | 1226 | 1561 | -296 | r | 335 | e | 39 | S |
| MIROC | RndFor | 1448 | 1802 | 1972 | 354 | e | 170 | e | 524 | E |
| MRI | BioClim | 1007 | 1910 | 2135 | 903 | e | 225 | e | 1128 | E |
| MRI | ENFA | 693 | 2786 | 2819 | 2093 | e | 33 | s | 2126 | E |
| MRI | EuclidDist | 1595 | 2125 | 2112 | 530 | e | -13 | s | 517 | E |
| MRI | FDA | 211 | 1833 | 1744 | 1622 | e | -89 | s | 1533 | E |
| MRI | GAM | 51 | 1879 | 1739 | 1828 | e | -140 | r | 1688 | E |
| MRI | GBM | 116 | 1625 | 1585 | 1509 | e | -40 | s | 1469 | E |
| MRI | GLM | 41 | 1816 | 1690 | 1775 | e | -126 | r | 1649 | E |
| MRI | GowerDist | 784 | 2018 | 1940 | 1234 | e | -78 | s | 1156 | E |
| MRI | MahalDist | 1201 | 2364 | 2153 | 1163 | e | -211 | r | 952 | E |
| MRI | MARS | 58 | 1686 | 1642 | 1628 | e | -44 | s | 1584 | E |
| MRI | MaxEnt | 52 | 1518 | 1513 | 1466 | e | -5 | s | 1461 | E |
| MRI | NNet | 155 | 2036 | 1657 | 1881 | e | -379 | r | 1502 | E |
| MRI | RndFor | 591 | 1792 | 1695 | 1201 | e | -97 | s | 1104 | E |

Legend:

AOGCM: coupled atmosphere-ocean general circulation models (for details, see Table S9);

ENM: algorithms (for details, Table S10);

Range size: geographical range size predicted by each combination of algorithm and AOGCM in three time periods (LGM – 21 ka, mid-Holocene – 6 ka, present-day – 0 ka). Unit is number of grid cells (0.5^o^ resolution) climatically suitable for *T. aurea*;

Range shift: "shift" – difference of range size between time periods (21-6 ka; 6-0 ka; 21-0 ka);

*"H"* – hypothesis from map classification matching the scenarios range expansion ("e"; shift > 110), range retraction ("r"; shift < -110), or range stability ("s"; -110 < shift < 110).

**Table S3.** Demographic hypotheses resulting from the palaeodistribution dynamics of *Tabebuia aurea* as predicted by the 52 maps (13 ENMs * 4 AOGCMs) obtained from the ecological niche modelling. The frequency represents the number of models (and percentage) matching each scenario previously established in Table S5 for two time slices (21-6ka and 6-0ka). The column "match 21-0ka" expresses the frequency of each scenario classified from LGM matching the previous classification considering the mid-Holocene predictions.

| **Map classification** | | **Frequency (%)** | **Match**  **21 – 0 ka** | **Demographic hypotheses** |
| --- | --- | --- | --- | --- |
| **21 - 6 ka** | **6 – 0 ka** |
| Expansion | Expansion | 6 (11.5) | Expansion (6/6)  Retraction (-)  Stability (-) | Range Expansion 21 - 0 ka |
| Expansion | Retraction | 4 (7.7) | Expansion (4/4)  Retraction (-)  Stability (-) | Range Stability |
| Expansion | Stability | 14 (27.0) | Expansion (14/14)  Retraction (-)  Stability (-) | Range Expansion 21- 6 ka |
| Retraction | Expansion | 19 (36.5) | Expansion (3/19)  Retraction (2/19)  Stability (14/19) | Range Expansion 6 - 0 ka |
| Retraction | Retraction | 0 (0.0) | - | - |
| Retraction | Stability | 1 (1.9) | Expansion (-)  Retraction (-)  Stability (1/1) | Range Retraction 21 - 6 ka |
| Stability | Expansion | 5 (9.6) | Expansion (5/5)  Retraction (-)  Stability (-) | Range Expansion 21- 0 ka |
| Stability | Retraction | 0 (0.0) | - | - |
| Stability | Stability | 3 (5.8) | Expansion (2/3)  Retraction (-)  Stability (1/3) | Range Stability |

**Table S4.** Proportion of membership (*Q*) of each pre-defined population of *Tabebuia aurea* in Central Brazil in each of the 19 clusters inferred by Bayesian analyses implemented in STRUCTURE software. N, number of individuals sampled in each population. In bold, Q ≥ 0.100.

|  | **Inferred Cluster** | | | | | | | | | | | | | | | | | | |  |
| --- | --- | --- | --- | --- | --- | --- | --- | --- | --- | --- | --- | --- | --- | --- | --- | --- | --- | --- | --- | --- |
| **Population** | **1** | **2** | **3** | **4** | **5** | **6** | **7** | **8** | **9** | **10** | **11** | **12** | **13** | **14** | **15** | **16** | **17** | **18** | **19** | **N** |
| **AGE** | 0.015 | 0.011 | 0.067 | 0.047 | 0.015 | 0.051 | 0.03 | 0.007 | 0.011 | 0.014 | 0.024 | 0.021 | 0.014 | 0.009 | 0.013 | 0.016 | **0.602** | 0.016 | 0.017 | 30 |
| **ARA** | **0.124** | 0.053 | **0.121** | 0.057 | 0.072 | 0.008 | 0.011 | 0.008 | **0.378** | 0.028 | 0.007 | 0.020 | 0.018 | 0.016 | 0.005 | 0.008 | 0.007 | 0.008 | 0.051 | 12 |
| **BAG** | 0.015 | 0.01 | 0.047 | 0.024 | 0.017 | 0.008 | 0.053 | 0.029 | 0.052 | 0.02 | 0.009 | 0.016 | 0.008 | 0.007 | 0.006 | 0.023 | 0.015 | 0.014 | **0.626** | 26 |
| **BAR** | **0.366** | 0.039 | 0.056 | 0.061 | 0.034 | 0.011 | 0.018 | 0.006 | **0.175** | 0.017 | 0.018 | 0.069 | 0.01 | 0.005 | 0.008 | 0.06 | 0.016 | 0.017 | 0.015 | 15 |
| **BOD** | 0.011 | 0.014 | 0.044 | 0.05 | 0.011 | **0.566** | 0.01 | 0.014 | 0.06 | 0.031 | 0.02 | 0.024 | 0.008 | 0.029 | 0.016 | 0.051 | 0.008 | 0.026 | 0.008 | 22 |
| **CAC** | 0.019 | 0.014 | 0.033 | 0.037 | 0.011 | 0.027 | 0.022 | 0.049 | 0.012 | **0.171** | 0.015 | **0.397** | 0.059 | 0.016 | 0.011 | 0.028 | 0.016 | 0.016 | 0.046 | 30 |
| **CHG** | 0.029 | 0.024 | 0.032 | 0.078 | 0.008 | 0.048 | 0.027 | 0.022 | 0.018 | **0.125** | 0.011 | 0.034 | **0.444** | 0.029 | 0.006 | 0.011 | 0.012 | 0.032 | 0.010 | 24 |
| **FAT** | **0.262** | 0.041 | 0**.124** | 0.043 | 0.019 | 0.031 | 0.044 | 0.008 | **0.161** | 0.027 | 0.012 | 0.018 | 0.017 | 0.014 | 0.006 | 0.008 | 0.016 | **0.138** | 0.011 | 35 |
| **GSV** | 0.012 | 0.025 | 0.052 | 0.021 | **0.521** | 0.02 | 0.026 | 0.031 | 0.021 | 0.022 | 0.058 | 0.018 | 0.009 | 0.032 | 0.025 | 0.056 | 0.027 | 0.012 | 0.012 | 8 |
| **PAN** | 0.064 | 0.047 | 0.054 | **0.141** | 0.023 | 0.062 | 0.019 | 0.013 | 0.081 | **0.123** | 0.015 | 0.044 | 0.055 | 0.021 | 0.031 | 0.055 | 0.018 | 0.029 | **0.103** | 23 |
| **POT** | 0.005 | 0.005 | 0.044 | 0.023 | 0.006 | 0.013 | 0.011 | 0.008 | 0.008 | 0.006 | 0.007 | 0.009 | 0.008 | **0.765** | 0.007 | 0.056 | 0.009 | 0.004 | 0.006 | 16 |
| **PNE** | 0.007 | 0.044 | 0.043 | 0.042 | 0.022 | **0.218** | 0.005 | 0.008 | 0.009 | 0.082 | 0.047 | 0.010 | 0.015 | 0.040 | 0.033 | **0.343** | 0.018 | 0.007 | 0.008 | 16 |
| **SDO** | **0.129** | 0.019 | 0.089 | **0.101** | 0.006 | 0.031 | 0.019 | **0.119** | 0.012 | **0.220** | 0.009 | 0.006 | 0.027 | 0.005 | 0.004 | **0.101** | 0.030 | 0.056 | 0.018 | 3 |
| **SEC** | 0.051 | 0.023 | **0.216** | 0.026 | 0.031 | 0.012 | 0.008 | **0.101** | **0.391** | 0.003 | 0.011 | 0.014 | 0.031 | 0.003 | 0.010 | 0.004 | 0.011 | 0.049 | 0.003 | 4 |
| **SUM** | 0.011 | 0.031 | 0.026 | 0.059 | 0.007 | 0.008 | 0.007 | **0.719** | 0.011 | 0.013 | 0.008 | 0.020 | 0.011 | 0.011 | 0.004 | 0.017 | 0.005 | 0.016 | 0.015 | 14 |
| **VIB** | 0.012 | **0.724** | 0.010 | 0.011 | 0.035 | 0.012 | 0.021 | 0.019 | 0.017 | 0.054 | 0.009 | 0.009 | 0.005 | 0.005 | 0.004 | 0.007 | 0.008 | 0.022 | 0.015 | 30 |
| **STZ** | 0.065 | 0.008 | 0.028 | 0.013 | 0.012 | 0.009 | **0.557** | 0.006 | 0.027 | 0.009 | 0.008 | 0.006 | 0.008 | 0.003 | 0.006 | 0.005 | 0.011 | **0.198** | 0.023 | 17 |
| **NIQ** | **0.110** | 0.021 | 0.022 | 0.017 | 0.017 | 0.011 | 0.083 | 0.011 | 0.026 | 0.063 | 0.054 | 0.011 | 0.010 | 0.005 | 0.009 | 0.007 | 0.030 | **0.483** | 0.010 | 28 |
| **SCA** | 0.006 | 0.007 | 0.007 | 0.008 | 0.006 | 0.012 | 0.006 | 0.027 | 0.007 | 0.005 | 0.013 | 0.009 | 0.006 | 0.005 | **0.836** | 0.018 | 0.007 | 0.01 | 0.005 | 29 |
| **PTU** | 0.022 | 0.008 | 0.049 | 0.037 | 0.036 | 0.042 | 0.034 | 0.023 | 0.012 | 0.010 | **0.408** | 0.008 | 0.012 | 0.023 | **0.135** | 0.032 | 0.036 | 0.041 | 0.032 | 32 |

**Table S5.** Number of migrants per generation (*N_e_m* ) for 20 populations of *Tabebuia aurea* in Brazil, based on Bayesian coalescence analysis. Migration direction is from populations in the columns into populations in the rows. In bold, values of *N_e_m* < 1.00

| **Population** | **AGE** | **ARA** | **BAG** | **BAR** | **BOD** | **CAC** | **CHG** | **FAT** | **GSV** | **PAN** | **POT** | **PNE** | **SDO** | **SEC** | **SUM** | **VIB** | **STZ** | **NIQ** | **SCA** | **PTU** |
| --- | --- | --- | --- | --- | --- | --- | --- | --- | --- | --- | --- | --- | --- | --- | --- | --- | --- | --- | --- | --- |
| **AGE** | 0.00 | 1.55 | 1.02 | 2.47 | 1.75 | 2.49 | 2.37 | 1.39 | 1.59 | 1.36 | 1.57 | 1.54 | 2.01 | 1.68 | 2.19 | 1.57 | 1.95 | 1.60 | 1.39 | 2.09 |
| **ARA** | 1.74 | 0.00 | 2.09 | 1.17 | 2.55 | 2.58 | 1.91 | 2.47 | 2.35 | 2.25 | 1.55 | 1.75 | 1.96 | 1.87 | 1.68 | 1.90 | 1.40 | 2.07 | 1.56 | 2.65 |
| **BAG** | 1.84 | 1.51 | 0.00 | 1.37 | 1.40 | 1.75 | 1.79 | 1.23 | 2.25 | 2.07 | 1.39 | 1.16 | 1.80 | 1.37 | 1.60 | 1.28 | 2.94 | 1.57 | 1.69 | 2.16 |
| **BAR** | 2.17 | 1.89 | 2.16 | 0.00 | 1.84 | 2.36 | 2.53 | 2.79 | 1.83 | 1.67 | 2.36 | 2.36 | 2.16 | 2.80 | 2.73 | 2.31 | 2.07 | 2.15 | 2.35 | 1.79 |
| **BOD** | 3.57 | 3.62 | 4.46 | 3.24 | 0.00 | 3.57 | 2.38 | 3.57 | 2.74 | 3.08 | 3.22 | 3.94 | 3.09 | 2.47 | 1.96 | 3.22 | 3.32 | 2.55 | 2.67 | 2.95 |
| **CAC** | 1.92 | 2.39 | 2.07 | 2.19 | 1.29 | 0.00 | 1.66 | 1.88 | 2.93 | 2.21 | 1.52 | 1.62 | 3.01 | 2.59 | 1.55 | 2.37 | 1.57 | 1.90 | 1.63 | 1.44 |
| **CHG** | 1.89 | 2.22 | 1.25 | 1.59 | 2.27 | 2.18 | 0.00 | 1.46 | 1.56 | 1.93 | 2.30 | 1.23 | 2.20 | 1.30 | 1.28 | 2.34 | 1.86 | 1.12 | 1.24 | 1.91 |
| **FAT** | 1.90 | 2.47 | 1.37 | 1.70 | 2.09 | 1.60 | 1.58 | 0.00 | 1.84 | 1.62 | 1.59 | 1.45 | 1.41 | 2.33 | 1.50 | 1.25 | 2.50 | 1.26 | 1.46 | 1.30 |
| **GSV** | 2.52 | 3.45 | 2.12 | 3.02 | 3.73 | 4.18 | 4.88 | 2.75 | 0.00 | 2.67 | 3.99 | 2.97 | 1.87 | 2.32 | 2.70 | 4.07 | 3.32 | 2.47 | 2.64 | 3.15 |
| **PAN** | 2.99 | 4.03 | 1.92 | 1.97 | 2.99 | 3.64 | 3.57 | 2.43 | 3.01 | 0.00 | 4.01 | 3.15 | 1.93 | 4.04 | 3.67 | 2.29 | 3.22 | 3.01 | 3.40 | 2.19 |
| **POT** | 2.02 | 1.26 | 2.39 | 2.06 | 1.92 | 1.80 | 1.09 | 2.14 | 1.49 | 1.85 | 0.00 | 1.40 | 1.99 | 1.57 | 2.07 | 1.19 | 1.56 | 1.54 | 1.79 | 2.21 |
| **PNE** | 2.18 | 4.67 | 2.62 | 5.73 | 2.84 | 2.90 | 3.18 | 3.40 | 2.66 | 2.38 | 3.20 | 0.00 | 4.74 | 2.19 | 3.42 | 3.08 | 2.72 | 3.62 | 2.60 | 3.31 |
| **SDO** | 14.09 | 9.02 | 14.71 | 12.36 | 15.36 | 21.41 | 16.05 | 10.59 | 20.08 | 17.05 | 22.14 | 15.50 | 0.00 | 20.44 | 20.88 | 15.06 | 16.96 | 22.86 | 9.24 | 11.38 |
| **SEC** | 2.76 | 4.10 | 3.51 | 1.84 | 5.16 | 2.95 | 3.30 | 2.67 | 2.85 | 2.51 | 3.07 | 3.43 | 5.09 | 0.00 | 3.52 | 2.68 | 3.36 | 5.70 | 3.89 | 3.07 |
| **SUM** | 1.41 | 0.97 | 1.01 | 1.36 | 1.45 | 1.25 | 1.27 | 1.28 | 2.11 | 1.84 | 1.47 | 1.19 | 1.22 | 1.55 | 0.00 | 1.00 | 1.29 | 1.51 | 1.62 | 0.86 |
| **VIB** | **0.90** | **0.85** | 1.24 | 1.53 | **0.64** | 1.08 | 1.35 | 1.50 | 1.40 | **0.92** | 1.32 | 1.00 | **0.91** | **0.90** | 1.17 | 0.00 | 1.49 | **0.93** | 1.06 | **0.96** |
| **STZ** | 2.27 | 3.21 | 1.56 | 2.00 | 1.48 | 2.17 | 2.33 | 1.98 | 2.60 | 2.08 | 2.42 | 2.28 | 1.66 | 2.55 | 2.81 | 1.58 | 0.00 | 2.23 | 1.30 | 1.65 |
| **NIQ** | 1.36 | **0.86** | 1.18 | 2.13 | 1.18 | **0.85** | 1.45 | 1.26 | 1.80 | 1.36 | 1.66 | **0.94** | 1.32 | 1.43 | 1.48 | 1.61 | 1.19 | 0.00 | **0.86** | **0.73** |
| **SCA** | **0.93** | 1.14 | 1.00 | **0.81** | **0.86** | **0.71** | **0.82** | **0.68** | **0.52** | 1.03 | **0.80** | 1.05 | 1.07 | 1.00 | **0.91** | 1.00 | 1.03 | 1.20 | 0.00 | 1.21 |
| **PTU** | 1.40 | 2.03 | 1.38 | 2.30 | 1.93 | 1.32 | 1.88 | 1.64 | **0.95** | 1.17 | 1.69 | 2.49 | 2.08 | 1.31 | 1.75 | 2.04 | 1.61 | 1.36 | 2.11 | 0.00 |

**Table S6.** Credibility interval (95%) of the number of migrants per generation (see Table S7 for the number of migrants) for 20 populations of *Tabebuia aurea* in Brazil, based on Bayesian coalescence analysis. Credibility interval for migration is from populations in the columns into populations in the rows.

|  |  | **AGE** |  | **ARA** |  | **BAG** |  | **BAR** |  | **BOD** |  | **CAC** |  | **CHG** |  | **FAT** |  | **GSV** |  | **PAN** |  | **POT** |  | **PNE** |  | **SDO** |  | **SEC** |  | **SUM** |  | **VIB** |  | **STZ** |  | **NIQ** |  | **SCA** |  | **PTU** |
| --- | --- | --- | --- | --- | --- | --- | --- | --- | --- | --- | --- | --- | --- | --- | --- | --- | --- | --- | --- | --- | --- | --- | --- | --- | --- | --- | --- | --- | --- | --- | --- | --- | --- | --- | --- | --- | --- | --- | --- | --- |
| AGE | 0.0000 | 0.0000 | 0.0003 | 4.3102 | 0.0005 | 5.7359 | 0.0003 | 5.8962 | 0.0005 | 13.3438 | 0.0340 | 140.4700 | 0.0003 | 4.0482 | 0.0007 | 4.5721 | 0.0010 | 6.4289 | 0.2964 | 7.1291 | 0.0002 | 6.4570 | 0.0008 | 5.8709 | 0.0067 | 53.4962 | 0.0005 | 9.3892 | 0.0002 | 3.6920 | 0.0002 | 2.8422 | 0.0004 | 6.2898 | 0.0002 | 4.7047 | 0.0003 | 3.1219 | 0.0012 | 4.6586 |
| ARA | 0.0364 | 3.0102 | 0.0000 | 0.0000 | 0.0003 | 4.5569 | 0.0998 | 4.7097 | 0.0007 | 9.1457 | 0.0107 | 246.0940 | 0.1065 | 6.1913 | 0.0004 | 4.5513 | 0.0004 | 7.2758 | 0.0011 | 7.8091 | 0.0007 | 3.3521 | 0.6547 | 11.5457 | 0.0022 | 21.6788 | 0.0005 | 12.7559 | 0.0002 | 2.6596 | 0.0002 | 2.1226 | 0.0012 | 5.7813 | 0.0003 | 2.3898 | 0.0019 | 2.7848 | 0.1752 | 4.4828 |
| BAG | 0.0004 | 2.5019 | 0.0003 | 4.7582 | 0.0000 | 0.0000 | 0.0126 | 5.4158 | 0.0005 | 12.8962 | 4.8861 | 180.7640 | 0.0002 | 3.5339 | 0.0003 | 4.5408 | 0.0005 | 4.6976 | 0.0006 | 7.1206 | 0.0031 | 4.9813 | 0.0004 | 6.4862 | 0.4936 | 42.3428 | 0.0007 | 8.9880 | 0.0002 | 2.2695 | 0.0002 | 3.0164 | 0.0004 | 4.6023 | 0.0002 | 3.1189 | 0.0002 | 2.6126 | 0.0003 | 3.4447 |
| BAR | 0.5603 | 5.6032 | 0.0003 | 2.8193 | 0.0003 | 3.8726 | 0.0000 | 0.0000 | 0.0004 | 8.1451 | 0.0124 | 225.0720 | 0.0003 | 3.2795 | 0.0004 | 5.5801 | 0.0007 | 9.7721 | 0.0005 | 5.9892 | 0.0003 | 5.9681 | 0.0742 | 17.2474 | 0.0052 | 44.9110 | 0.0005 | 6.6221 | 0.0002 | 3.4945 | 0.2002 | 3.1550 | 0.0004 | 4.1568 | 0.0002 | 4.6648 | 0.0001 | 2.9640 | 0.4804 | 4.6870 |
| BOD | 0.0003 | 5.4986 | 0.0006 | 5.7063 | 0.0002 | 4.1664 | 0.0007 | 4.5007 | 0.0000 | 0.0000 | 5.5356 | 131.7240 | 0.1056 | 5.8437 | 0.0147 | 4.6923 | 0.0022 | 8.4354 | 0.0201 | 11.9399 | 0.0004 | 4.7820 | 0.0005 | 7.7341 | 0.0036 | 51.2049 | 0.0013 | 10.7958 | 0.0003 | 5.0784 | 0.0002 | 1.7427 | 0.0344 | 3.6974 | 0.0003 | 2.4035 | 0.0001 | 2.2067 | 0.0021 | 3.7817 |
| CAC | 0.0008 | 8.8290 | 0.0003 | 5.5817 | 0.0052 | 3.5478 | 0.2172 | 5.1306 | 0.3729 | 8.1366 | 0.0000 | 0.0000 | 0.0003 | 5.3454 | 0.0006 | 3.7398 | 0.0007 | 12.8397 | 0.0005 | 8.9153 | 0.0002 | 4.2730 | 0.0664 | 8.4385 | 1.8252 | 68.9259 | 0.0008 | 11.1415 | 0.0010 | 3.2045 | 0.0008 | 3.3253 | 0.0009 | 5.0712 | 0.0003 | 2.1100 | 0.0002 | 1.6215 | 0.0004 | 4.2877 |
| CHG | 0.0007 | 6.6359 | 0.0006 | 5.4176 | 0.0395 | 4.2564 | 0.0270 | 6.0211 | 0.0006 | 5.5164 | 0.0101 | 154.0520 | 0.0000 | 0.0000 | 0.0002 | 3.5041 | 0.0010 | 13.7063 | 0.4041 | 7.0299 | 0.0002 | 3.5189 | 0.0005 | 7.2024 | 0.0036 | 30.0647 | 0.0018 | 9.5472 | 0.0019 | 2.4363 | 0.0002 | 3.1107 | 0.0068 | 6.2719 | 0.0004 | 3.0486 | 0.0001 | 2.0139 | 0.0997 | 3.7580 |
| FAT | 0.0003 | 3.1721 | 0.0025 | 5.0120 | 0.0009 | 3.5343 | 0.0005 | 6.7442 | 0.0005 | 7.9679 | 11.2990 | 153.7170 | 0.0003 | 5.1936 | 0.0000 | 0.0000 | 0.0005 | 7.4635 | 0.0005 | 7.2686 | 0.0005 | 6.5270 | 0.0005 | 9.6654 | 0.0024 | 29.0413 | 0.0010 | 7.5754 | 0.0218 | 2.7852 | 0.0004 | 3.1505 | 0.0005 | 8.5179 | 0.0002 | 2.9879 | 0.0001 | 1.5709 | 0.0003 | 4.4733 |
| GSV | 0.0005 | 4.6542 | 0.0021 | 6.1657 | 0.0036 | 6.9849 | 0.0004 | 6.4819 | 0.3190 | 6.0459 | 30.4010 | 207.2950 | 0.0004 | 3.8809 | 0.0003 | 4.7812 | 0.0000 | 0.0000 | 0.6161 | 6.6176 | 0.0003 | 2.7908 | 0.0006 | 6.8286 | 0.7307 | 42.6460 | 0.0005 | 7.7220 | 0.0005 | 4.6379 | 0.0374 | 2.6366 | 0.0006 | 6.3917 | 0.1698 | 4.3199 | 0.0001 | 1.4282 | 0.0003 | 2.6574 |
| PAN | 0.0003 | 4.0572 | 0.0007 | 8.0358 | 0.0067 | 6.9564 | 0.0004 | 5.7066 | 0.0004 | 7.9706 | 0.0498 | 282.3490 | 0.0004 | 4.5447 | 0.0005 | 5.2370 | 0.0629 | 6.3234 | 0.0000 | 0.0000 | 0.0003 | 4.1439 | 0.0005 | 6.4157 | 0.3888 | 39.8852 | 0.0005 | 7.1154 | 0.0002 | 4.2845 | 0.0003 | 2.2529 | 0.0008 | 5.8868 | 0.0003 | 2.6788 | 0.0002 | 2.7932 | 0.0002 | 3.3091 |
| POT | 0.0003 | 3.2081 | 0.0004 | 5.2322 | 0.0004 | 6.4942 | 0.0004 | 5.4346 | 0.0451 | 11.6902 | 0.0106 | 183.6920 | 0.0003 | 4.2823 | 0.0003 | 4.8465 | 0.1004 | 12.3684 | 0.0008 | 11.3564 | 0.0000 | 0.0000 | 0.0005 | 8.9336 | 0.0032 | 80.7976 | 0.0008 | 8.0556 | 0.0002 | 3.7105 | 0.0508 | 4.2534 | 0.0007 | 8.3147 | 0.1399 | 3.2900 | 0.0003 | 1.7884 | 0.0004 | 4.8374 |
| PNE | 0.0002 | 5.1992 | 0.0009 | 3.3983 | 0.0002 | 3.0615 | 0.0009 | 4.8736 | 0.0528 | 9.7578 | 0.1104 | 137.7690 | 0.0002 | 2.7709 | 0.0004 | 3.4159 | 0.0657 | 5.8200 | 0.0028 | 8.3999 | 0.0003 | 4.7942 | 0.0000 | 0.0000 | 0.0111 | 33.4127 | 0.0005 | 10.4231 | 0.0004 | 2.7649 | 0.0324 | 1.9593 | 0.0015 | 4.7777 | 0.0312 | 3.0573 | 0.0001 | 2.1867 | 0.2333 | 4.5932 |
| SDO | 0.0323 | 8.6334 | 0.0127 | 3.6853 | 0.0004 | 4.1462 | 0.0007 | 3.9215 | 0.2871 | 7.4489 | 0.0145 | 251.7330 | 0.0005 | 6.3311 | 0.0002 | 3.4866 | 0.0005 | 5.7846 | 0.0006 | 6.0188 | 0.0009 | 6.6543 | 0.6422 | 9.4846 | 0.0000 | 0.0000 | 0.0416 | 14.2684 | 0.0004 | 2.5061 | 0.0005 | 1.8389 | 0.1048 | 4.6392 | 0.0120 | 3.1515 | 0.0001 | 2.5882 | 0.0069 | 5.5930 |
| SEC | 0.0005 | 3.8906 | 0.0039 | 4.2351 | 0.0002 | 6.5293 | 0.4531 | 5.7265 | 0.0004 | 7.0389 | 0.0109 | 231.6990 | 0.0002 | 2.7129 | 0.6813 | 4.3084 | 0.0004 | 6.5989 | 0.0004 | 11.1009 | 0.2194 | 2.8743 | 0.0006 | 4.6005 | 0.0033 | 65.8295 | 0.0000 | 0.0000 | 0.0003 | 7.9661 | 0.0002 | 1.9323 | 0.0011 | 5.1612 | 0.0302 | 3.4574 | 0.0001 | 1.8378 | 0.0007 | 4.6495 |
| SUM | 0.0004 | 5.8020 | 0.0436 | 6.2388 | 0.1346 | 3.4724 | 0.0015 | 8.5661 | 0.0005 | 9.9217 | 1.5128 | 152.3740 | 0.0008 | 3.1934 | 0.0003 | 3.0873 | 0.0011 | 9.6352 | 0.0004 | 8.9947 | 0.0524 | 4.8211 | 0.0007 | 8.0743 | 0.0064 | 50.8515 | 0.0007 | 10.5193 | 0.0000 | 0.0000 | 0.0005 | 2.3049 | 0.0014 | 5.9196 | 0.0002 | 3.0377 | 0.0001 | 2.3591 | 0.0004 | 4.1406 |
| VIB | 0.0002 | 3.4015 | 0.0004 | 4.3495 | 0.0047 | 2.8705 | 0.0008 | 5.4368 | 0.0005 | 8.3040 | 0.0103 | 256.5870 | 0.0003 | 5.3292 | 0.0002 | 3.5234 | 0.0004 | 10.0475 | 0.0006 | 4.7117 | 0.0002 | 3.8575 | 0.0006 | 13.9560 | 0.0040 | 35.2695 | 0.0007 | 7.1676 | 0.0002 | 2.6430 | 0.0000 | 0.0000 | 0.0003 | 3.9573 | 0.0672 | 3.7703 | 0.0004 | 2.2296 | 0.0023 | 6.3067 |
| STZ | 0.4390 | 3.9751 | 0.0003 | 3.1513 | 0.0003 | 5.8091 | 0.0004 | 4.3465 | 0.0005 | 7.6349 | 0.0110 | 197.5030 | 0.0022 | 5.5184 | 0.0007 | 6.1833 | 0.0004 | 10.1650 | 0.5741 | 7.4175 | 0.0004 | 3.3404 | 0.0008 | 6.4665 | 0.0040 | 57.0792 | 0.1387 | 8.5390 | 0.0002 | 3.6818 | 0.0005 | 3.8865 | 0.0000 | 0.0000 | 0.0002 | 4.1171 | 0.0002 | 2.8342 | 0.0003 | 4.9050 |
| NIQ | 0.0022 | 4.0305 | 0.0004 | 5.9782 | 0.2574 | 4.8312 | 0.0244 | 6.3525 | 0.0267 | 6.3734 | 0.0224 | 176.4530 | 0.0004 | 3.7806 | 0.0003 | 3.5587 | 0.0008 | 6.3840 | 0.0005 | 7.4164 | 0.0003 | 3.7888 | 0.0010 | 12.8836 | 0.1405 | 59.7134 | 0.0006 | 19.9161 | 0.0005 | 3.1802 | 0.0002 | 2.3870 | 0.0014 | 7.3357 | 0.0000 | 0.0000 | 0.0511 | 2.7788 | 0.0002 | 3.4369 |
| SCA | 0.0004 | 3.4135 | 0.0003 | 3.8921 | 0.0004 | 3.6579 | 0.0924 | 7.4565 | 0.0013 | 6.1409 | 0.0479 | 152.1730 | 0.0002 | 3.0048 | 0.0003 | 3.9994 | 0.0004 | 5.8398 | 0.0031 | 6.8184 | 0.0144 | 3.1299 | 0.0008 | 5.7052 | 0.0025 | 28.3080 | 0.0025 | 8.8628 | 0.0002 | 2.9297 | 0.0002 | 3.2059 | 0.0005 | 3.7389 | 0.0002 | 1.8836 | 0.0000 | 0.0000 | 0.0004 | 5.5794 |
| PTU | 0.0006 | 4.6106 | 0.0036 | 8.5789 | 0.0003 | 5.2022 | 0.0004 | 4.0801 | 0.0004 | 6.9243 | 0.0114 | 165.1070 | 0.0003 | 4.0215 | 0.0020 | 2.8955 | 0.6477 | 6.2364 | 0.0005 | 5.6377 | 0.0003 | 8.2029 | 0.0005 | 12.2838 | 0.0025 | 40.1880 | 0.0005 | 6.3563 | 0.0004 | 3.2285 | 0.0027 | 3.3256 | 0.0020 | 3.7622 | 0.0002 | 2.3598 | 0.0001 | 3.3089 | 0.0000 | 0.0000 |

|  | **CMT** | | **ABMT** | | **PGO** | | **SMS** | | **AMS** | | **ATO** | | **SMGO** | | **LGO** | | **ISP** | | **MAMG** | | **ENGO** | | **STGO** | | **AMG** | | **PMG** | | **PMS** | | **PCMS** | | **CMS** | | **IGO** | | **RAMT** | | **RAGO** | | **JGO** | | **NTO** | | **ARTO** | | **AQMS** | | **CAMT** | |
| --- | --- | --- | --- | --- | --- | --- | --- | --- | --- | --- | --- | --- | --- | --- | --- | --- | --- | --- | --- | --- | --- | --- | --- | --- | --- | --- | --- | --- | --- | --- | --- | --- | --- | --- | --- | --- | --- | --- | --- | --- | --- | --- | --- | --- | --- | --- | --- | --- | --- | --- |
| **CMT** |  |  | 0.003 | 0.798 | 0.375 | 0.846 | 0.260 | 0.513 | 0.174 | 0.635 | 0.157 | 0.776 | 0.172 | 0.766 | 0.176 | 0.786 | 0.196 | 0.381 | 0.405 | 0.540 | 1.068 | 4.746 | 1.451 | 3.195 | 0.429 | 0.860 | 0.236 | 0.743 | 0.592 | 0.850 | 0.549 | 0.752 | 0.891 | 1.281 | 0.176 | 0.432 | 0.163 | 0.630 | 0.148 | 0.751 | 0.027 | 0.559 | 0.363 | 0.882 | 0.096 | 0.826 | 0.559 | 0.837 | 0.541 | 0.949 |
| **ABMT** | 0.003 | 0.427 |  |  | 0.669 | 0.961 | 0.830 | 1.243 | 0.693 | 0.988 | 0.215 | 0.634 | 0.173 | 0.682 | 0.255 | 0.762 | 0.005 | 0.693 | 0.403 | 0.731 | 0.182 | 0.610 | 0.169 | 0.739 | 0.212 | 0.733 | 0.375 | 1.007 | 0.326 | 0.753 | 0.489 | 1.564 | 0.219 | 0.846 | 0.299 | 0.833 | 0.520 | 2.376 | 0.381 | 1.260 | 0.294 | 0.845 | 0.005 | 0.991 | 0.339 | 1.006 | 0.335 | 0.950 | 0.225 | 0.806 |
| **PGO** | 0.278 | 0.931 | 1.025 | 2.229 |  |  | 0.023 | 0.629 | 0.088 | 0.664 | 0.045 | 0.543 | 0.120 | 0.619 | 0.001 | 0.518 | 0.580 | 0.857 | 0.192 | 0.508 | 0.658 | 1.251 | 0.024 | 0.814 | 0.165 | 0.860 | 0.285 | 0.890 | 0.002 | 0.361 | 0.316 | 0.930 | 0.010 | 0.273 | 0.020 | 0.399 | 0.543 | 1.875 | 0.403 | 1.778 | 0.454 | 0.963 | 0.763 | 1.520 | 0.913 | 1.572 | 0.902 | 1.371 | 0.455 | 0.750 |
| **SMS** | 0.453 | 0.890 | 0.653 | 0.973 | 0.156 | 0.829 |  |  | 0.093 | 0.676 | 0.475 | 0.730 | 0.078 | 0.468 | 0.251 | 0.864 | 0.288 | 0.848 | 0.089 | 0.664 | 0.401 | 0.976 | 0.017 | 0.094 | 0.204 | 0.266 | 0.138 | 0.338 | 0.163 | 0.350 | 0.290 | 0.752 | 0.290 | 0.760 | 0.195 | 0.443 | 0.317 | 0.992 | 0.178 | 0.847 | 0.121 | 0.454 | 0.000 | 0.313 | 0.112 | 0.668 | 0.700 | 1.757 | 0.204 | 0.697 |
| **AMS** | 0.179 | 0.616 | 0.523 | 1.334 | 0.110 | 0.660 | 0.548 | 1.399 |  |  | 0.009 | 0.099 | 0.496 | 1.008 | 0.269 | 0.865 | 0.154 | 0.764 | 0.126 | 0.538 | 0.163 | 0.796 | 0.240 | 0.860 | 0.106 | 0.499 | 0.423 | 0.947 | 0.330 | 0.719 | 0.579 | 1.577 | 0.378 | 0.981 | 0.684 | 1.229 | 0.850 | 1.727 | 0.598 | 0.960 | 0.280 | 0.702 | 0.112 | 0.687 | 0.002 | 0.178 | 0.243 | 0.726 | 0.207 | 0.930 |
| **ATO** | 0.153 | 0.982 | 0.207 | 0.809 | 0.144 | 0.564 | 0.186 | 0.416 | 0.404 | 0.845 |  |  | 0.018 | 0.266 | 0.287 | 0.901 | 0.150 | 0.550 | 0.258 | 0.730 | 0.828 | 1.878 | 0.495 | 0.960 | 0.202 | 0.943 | 0.124 | 0.443 | 0.129 | 0.446 | 0.200 | 0.964 | 0.588 | 1.004 | 0.088 | 0.379 | 0.303 | 1.068 | 0.134 | 0.726 | 0.583 | 1.128 | 0.730 | 2.498 | 0.179 | 0.629 | 0.599 | 0.923 | 0.127 | 0.804 |
| **SMGO** | 0.320 | 0.825 | 0.302 | 0.929 | 0.160 | 0.716 | 0.150 | 0.750 | 0.563 | 1.162 | 0.007 | 0.222 |  |  | 0.417 | 0.942 | 0.235 | 0.703 | 0.254 | 0.757 | 0.760 | 1.808 | 0.542 | 1.274 | 0.026 | 0.474 | 0.135 | 0.589 | 0.092 | 0.360 | 0.126 | 0.632 | 0.060 | 0.260 | 0.209 | 0.526 | 0.162 | 0.526 | 0.479 | 0.985 | 0.470 | 1.005 | 0.581 | 1.067 | 0.411 | 0.844 | 0.122 | 0.726 | 0.002 | 0.324 |
| **LGO** | 0.070 | 0.382 | 0.231 | 0.851 | 0.039 | 0.260 | 0.353 | 0.956 | 0.104 | 0.610 | 0.077 | 0.734 | 0.375 | 1.005 |  |  | 0.950 | 1.911 | 0.137 | 0.789 | 0.054 | 0.478 | 0.232 | 0.767 | 0.277 | 0.585 | 0.145 | 0.887 | 0.049 | 0.326 | 0.291 | 0.698 | 0.169 | 0.587 | 0.938 | 1.938 | 1.627 | 2.523 | 1.140 | 2.165 | 0.274 | 0.681 | 0.000 | 0.099 | 0.001 | 0.327 | 0.453 | 1.002 | 0.050 | 0.501 |
| **ISP** | 0.003 | 0.151 | 0.080 | 0.330 | 0.578 | 1.003 | 0.357 | 0.977 | 0.323 | 0.620 | 0.186 | 0.611 | 0.308 | 0.969 | 0.620 | 0.993 |  |  | 0.396 | 0.800 | 0.151 | 0.832 | 0.680 | 1.114 | 0.107 | 0.769 | 0.198 | 0.935 | 0.026 | 0.360 | 0.154 | 0.926 | 0.428 | 0.919 | 0.367 | 0.764 | 0.034 | 0.430 | 0.113 | 0.326 | 0.501 | 0.888 | 0.257 | 0.661 | 0.341 | 0.902 | 0.745 | 1.040 | 0.500 | 1.827 |
| **MAMG** | 0.208 | 0.860 | 0.170 | 0.584 | 0.330 | 0.664 | 0.019 | 0.565 | 0.074 | 0.502 | 0.388 | 0.722 | 0.323 | 0.958 | 0.344 | 0.970 | 0.186 | 0.881 |  |  | 0.223 | 0.988 | 0.051 | 0.522 | 0.001 | 0.245 | 0.591 | 2.022 | 0.013 | 0.392 | 0.130 | 0.977 | 0.172 | 0.926 | 0.111 | 0.805 | 0.142 | 0.643 | 0.019 | 0.360 | 0.121 | 0.970 | 0.519 | 0.990 | 0.378 | 0.928 | 0.003 | 0.223 | 0.131 | 0.758 |
| **ENGO** | 1.134 | 1.902 | 0.103 | 0.958 | 0.473 | 0.923 | 0.109 | 0.949 | 0.169 | 0.822 | 0.068 | 0.755 | 0.537 | 1.000 | 0.151 | 0.722 | 0.069 | 0.612 | 0.091 | 0.392 |  |  | 0.160 | 0.913 | 0.024 | 0.476 | 0.061 | 0.770 | 0.066 | 0.350 | 0.084 | 0.394 | 0.277 | 0.889 | 0.028 | 0.423 | 0.092 | 0.367 | 0.069 | 0.471 | 0.064 | 0.669 | 0.001 | 0.546 | 0.377 | 0.900 | 0.007 | 0.342 | 0.014 | 0.280 |
| **STGO** | 0.571 | 0.960 | 0.009 | 0.381 | 0.098 | 0.442 | 0.207 | 0.726 | 0.078 | 0.370 | 0.146 | 0.662 | 0.207 | 0.726 | 0.160 | 0.780 | 0.364 | 0.860 | 0.015 | 0.298 | 0.048 | 0.400 |  |  | 0.493 | 0.857 | 0.415 | 1.022 | 0.002 | 0.051 | 0.128 | 0.488 | 0.455 | 0.886 | 0.348 | 0.719 | 0.121 | 0.830 | 0.069 | 0.371 | 0.159 | 0.572 | 0.170 | 0.526 | 0.499 | 1.271 | 0.046 | 0.149 | 0.144 | 0.428 |
| **AMG** | 0.296 | 0.891 | 0.435 | 0.969 | 0.287 | 0.948 | 0.245 | 0.806 | 0.711 | 1.032 | 0.575 | 0.885 | 0.115 | 0.500 | 0.297 | 0.621 | 0.089 | 0.377 | 0.001 | 0.104 | 0.171 | 0.523 | 1.265 | 2.316 |  |  | 0.695 | 1.651 | 0.037 | 0.417 | 0.529 | 1.011 | 0.302 | 0.831 | 0.355 | 0.840 | 0.153 | 0.438 | 0.001 | 0.074 | 0.141 | 0.441 | 0.488 | 0.780 | 0.225 | 0.743 | 0.104 | 0.390 | 0.247 | 0.627 |
| **PMG** | 0.227 | 0.726 | 0.488 | 0.923 | 0.326 | 0.931 | 0.046 | 0.215 | 0.109 | 0.847 | 0.246 | 0.659 | 0.157 | 0.534 | 0.510 | 0.845 | 0.179 | 0.522 | 0.499 | 0.918 | 0.183 | 0.608 | 0.889 | 2.133 | 0.764 | 1.836 |  |  | 0.032 | 0.233 | 0.282 | 0.634 | 0.261 | 0.954 | 0.176 | 0.481 | 0.170 | 0.622 | 0.596 | 0.963 | 0.620 | 1.161 | 0.579 | 1.694 | 0.780 | 1.091 | 0.720 | 1.260 | 0.120 | 0.626 |
| **PMS** | 0.001 | 0.007 | 0.176 | 0.847 | 0.018 | 0.204 | 0.103 | 0.260 | 0.339 | 0.718 | 0.103 | 0.369 | 0.106 | 0.379 | 0.024 | 0.389 | 0.013 | 0.335 | 0.025 | 0.283 | 0.161 | 0.401 | 0.001 | 0.069 | 0.125 | 0.460 | 0.349 | 0.635 |  |  | 0.898 | 1.260 | 0.917 | 1.528 | 0.001 | 0.097 | 0.225 | 0.592 | 0.126 | 0.326 | 0.181 | 0.485 | 0.006 | 0.094 | 0.139 | 0.553 | 0.051 | 0.205 | 0.298 | 0.805 |
| **PCMS** | 0.539 | 1.303 | 0.556 | 0.998 | 0.264 | 0.869 | 0.148 | 0.801 | 0.180 | 0.913 | 0.286 | 0.861 | 0.682 | 1.042 | 0.173 | 0.367 | 0.181 | 0.411 | 0.242 | 0.538 | 0.029 | 0.119 | 0.231 | 0.831 | 0.372 | 0.812 | 0.147 | 0.292 | 0.225 | 0.732 |  |  | 0.535 | 1.386 | 0.217 | 0.536 | 0.212 | 0.511 | 0.388 | 0.734 | 0.159 | 0.613 | 0.021 | 0.116 | 0.282 | 0.808 | 0.150 | 0.749 | 0.119 | 0.360 |
| **CMS** | 0.733 | 0.962 | 0.330 | 0.860 | 0.030 | 0.197 | 0.255 | 0.729 | 0.128 | 0.753 | 0.329 | 0.713 | 0.109 | 0.438 | 0.147 | 0.524 | 0.234 | 0.611 | 0.198 | 0.586 | 0.018 | 0.106 | 0.069 | 0.199 | 0.296 | 0.764 | 0.348 | 0.828 | 0.526 | 0.878 | 0.843 | 2.544 |  |  | 0.085 | 0.206 | 0.082 | 0.226 | 0.102 | 0.226 | 0.517 | 0.959 | 0.433 | 0.946 | 0.468 | 0.940 | 0.331 | 0.862 | 0.153 | 0.485 |
| **IGO** | 0.161 | 0.488 | 0.228 | 0.854 | 0.085 | 0.215 | 0.208 | 0.876 | 0.601 | 1.141 | 0.104 | 0.589 | 0.492 | 0.910 | 0.911 | 1.295 | 0.033 | 0.118 | 0.198 | 0.719 | 0.139 | 0.613 | 0.637 | 1.586 | 0.166 | 0.742 | 0.239 | 0.745 | 0.045 | 0.158 | 0.269 | 0.845 | 0.014 | 0.104 |  |  | 0.677 | 1.907 | 0.710 | 1.234 | 0.324 | 0.826 | 0.122 | 0.473 | 0.224 | 0.703 | 0.582 | 1.260 | 0.447 | 0.957 |
| **RAMT** | 0.148 | 0.511 | 0.731 | 1.287 | 0.424 | 0.825 | 0.203 | 0.748 | 0.354 | 0.726 | 0.159 | 0.726 | 0.170 | 0.526 | 1.095 | 1.917 | 0.013 | 0.103 | 0.150 | 0.890 | 0.143 | 0.426 | 0.711 | 1.260 | 0.107 | 0.285 | 0.053 | 0.206 | 0.115 | 0.360 | 0.361 | 0.736 | 0.071 | 0.149 | 0.417 | 0.725 |  |  | 0.857 | 1.823 | 0.323 | 0.856 | 0.218 | 0.773 | 0.187 | 0.433 | 0.511 | 0.992 | 0.209 | 0.727 |
| **RAGO** | 0.131 | 0.766 | 0.306 | 0.729 | 0.314 | 0.978 | 0.688 | 0.948 | 0.130 | 0.588 | 0.152 | 0.664 | 0.355 | 0.852 | 1.231 | 2.268 | 0.510 | 0.831 | 0.084 | 0.260 | 0.287 | 0.737 | 0.486 | 0.907 | 0.015 | 0.153 | 0.046 | 0.148 | 0.082 | 0.207 | 0.728 | 1.793 | 0.017 | 0.194 | 0.789 | 1.498 | 0.969 | 2.576 |  |  | 0.350 | 0.740 | 0.170 | 0.312 | 0.257 | 0.418 | 0.264 | 0.610 | 0.213 | 0.746 |
| **JGO** | 0.184 | 0.602 | 0.391 | 0.924 | 0.469 | 1.009 | 0.243 | 0.683 | 0.263 | 0.760 | 0.397 | 0.639 | 0.488 | 0.939 | 0.125 | 0.996 | 0.475 | 0.847 | 0.151 | 0.329 | 0.108 | 0.448 | 0.317 | 0.931 | 0.218 | 0.805 | 0.214 | 0.394 | 0.317 | 0.665 | 0.214 | 1.126 | 0.936 | 1.640 | 0.506 | 0.826 | 0.889 | 1.632 | 1.290 | 2.362 |  |  | 0.467 | 0.805 | 0.795 | 1.017 | 0.050 | 0.118 | 0.403 | 0.913 |
| **NTO** | 0.480 | 0.913 | 0.072 | 0.195 | 0.344 | 0.817 | 0.003 | 0.143 | 0.109 | 0.659 | 0.653 | 0.963 | 0.177 | 0.536 | 0.003 | 0.101 | 0.076 | 0.310 | 0.206 | 0.332 | 0.091 | 0.377 | 0.521 | 0.930 | 0.157 | 0.526 | 0.450 | 1.083 | 0.495 | 0.957 | 0.013 | 0.107 | 0.397 | 0.826 | 0.070 | 0.204 | 0.149 | 0.495 | 0.263 | 0.500 | 0.291 | 0.626 |  |  | 0.383 | 0.769 | 0.496 | 0.975 | 0.354 | 0.989 |
| **ARTO** | 0.236 | 0.841 | 0.145 | 0.679 | 0.604 | 1.055 | 0.227 | 0.758 | 0.002 | 0.094 | 0.158 | 0.457 | 0.370 | 0.756 | 0.095 | 0.263 | 0.106 | 0.460 | 0.202 | 0.413 | 0.855 | 1.390 | 1.947 | 3.144 | 0.372 | 0.726 | 0.511 | 1.122 | 0.025 | 0.096 | 0.646 | 0.954 | 0.585 | 0.926 | 0.118 | 0.630 | 0.530 | 0.907 | 0.365 | 0.936 | 0.370 | 0.840 | 0.214 | 0.777 |  |  | 0.254 | 2.521 | 0.158 | 0.599 |
| **AQMS** | 0.109 | 0.608 | 0.058 | 0.203 | 0.226 | 0.633 | 0.581 | 1.364 | 0.255 | 0.373 | 0.257 | 0.895 | 0.118 | 0.526 | 0.633 | 1.567 | 0.756 | 0.970 | 0.105 | 0.318 | 0.017 | 0.341 | 0.272 | 0.438 | 0.047 | 0.329 | 0.105 | 0.217 | 0.824 | 1.433 | 0.796 | 1.833 | 0.858 | 1.503 | 0.262 | 0.626 | 0.642 | 0.951 | 0.683 | 0.971 | 0.565 | 0.864 | 0.103 | 0.230 | 0.141 | 0.320 |  |  | 0.588 | 0.841 |
| **CAMT** | 0.429 | 0.956 | 0.504 | 0.720 | 0.254 | 0.786 | 0.391 | 0.856 | 0.694 | 1.661 | 0.141 | 0.697 | 0.017 | 0.149 | 0.186 | 0.586 | 0.513 | 0.922 | 0.178 | 0.408 | 0.121 | 0.491 | 0.170 | 0.541 | 0.103 | 0.237 | 0.048 | 0.215 | 0.366 | 0.756 | 0.386 | 0.838 | 0.153 | 0.549 | 0.249 | 0.623 | 0.422 | 0.938 | 0.596 | 0.974 | 0.531 | 0.720 | 0.123 | 0.236 | 0.104 | 0.355 | 0.686 | 1.587 |  |  |

**Table S7.** Profile of genetic parameters obtained from the 2,000 simulations of six different demographic scenarios, using the software BayeSSC. *H_e_* – genetic diversity; *A* – total number of alleles; *Variance* – allelic size variance (for the first locus). Eleven loci were simulated. See Figure 5 for details about the demographical scenarios and simulations.

|  | ***H_e_*** | | ***A*** | | ***Variance*** | |
| --- | --- | --- | --- | --- | --- | --- |
| **Models** | ***Range*** | ***Mean (SD)*** | ***Range*** | ***Mean (SD)*** | ***Range*** | ***Mean (SD)*** |
| **Retraction 21 - 6 kyr BP** | 0.998 – 1.000 | 0.999 (0.001) | 420 - 769 | 543.23 (49.17) | 22.43 – 845.11 | 87.59 (88.11) |
| **Stability 21 - 0 kyr BP** | 0.988 – 0.994 | 0.993 (0.001) | 234 - 508 | 375.68 (45.68) | 10.18 – 695.92 | 62.63 (64.77) |
| **Expansion 21 - 0 kyr BP** | 0.599 – 0.937 | 0.885 (0.034) | 28 - 87 | 51.83 (6.68) | 0.02 – 4.02 | 0.48 (0.52) |
| **Expansion 6 - 0 kyr BP** | 0.562 – 0.940 | 0.884 (0.039) | 32 - 79 | 41.59 (4.04) | 0.01 – 5.98 | 0.35 (0.46) |
| **Expansion 21 - 6 kyr BP** | 0.382 – 0.917 | 0.771 (0.097) | 22 - 58 | 38.05 (5.81) | 0.001 – 1.04 | 0.12 (0.14) |
| **Multiple Refugia 21 - 0 kyr BP** | 0.371 – 0.929 | 0.769 (0.099) | 21 - 56 | 37.65 (5.80) | 0.001- 1.01 | 0.13 (0.14) |

**Table S8.** Details on the palaeoclimatic simulations (AOGCMs) used in the ecological niche modelling of *Tabebuia aurea.*

| **Model ID** | **Modelling Center** | **Resolution*** | **Source** | **Year** |
| --- | --- | --- | --- | --- |
| CCSM4 | University of Miami – RSMAS, USA | 0.9° × 1.25° | CMIP5/PMIP3 | 2012 |
| CNRM-CM5 | Centre National de Recherches Meteorologiques / Centre Europeen de Recherche et Formation Avancees en Calcul Scientifique, France | 1.4° x 1.4° | CMIP5/PMIP3 | 2012 |
| MIROC-ESM | Atmosphere and Ocean Research Institute (University of Tokyo), National Institute for Environmental Studies, and Japan Agency for Marine-Earth Science and Technology, Japan | 2.8° × 2.8° | CMIP5/PMIP3 | 2012 |
| MRI-CGCM3 | Meteorological Research Institute, Japan | 1.1° x 1.1° | CMIP5/PMIP3 | 2012 |

* longitude × latitude

CMIP5 – Coupled Model Intercomparison Project, Phase 5 (<http://cmip-pcmdi.llnl.gov/>)

PMIP3 – Paleoclimate Modelling Intercomparison Project, Phase 3 (<http://pmip3.lsce.ipsl.fr/>)

**Table S9**. Loadings of the bioclimatic variables in the first five axes of Varimax Rotated Factor Analysis, based on the AOGCM CCSM. Numbers in bold highlight the highest loading, and based on this highest value one variable per factor was selected.

| **Bioclimatic variables** | **I** | **II** | **III** | **IV** | **V** |
| --- | --- | --- | --- | --- | --- |
| **1** | **0.967** | 0.088 | 0.158 | 0.154 | 0.096 |
| 2 | 0.024 | -0.027 | 0.288 | -0.835 | 0.290 |
| 3 | 0.330 | 0.275 | 0.142 | 0.178 | 0.529 |
| 4 | -0.352 | -0.345 | -0.087 | -0.655 | -0.489 |
| 5 | 0.942 | 0.062 | 0.207 | -0.237 | -0.047 |
| 6 | 0.861 | 0.165 | 0.080 | 0.460 | 0.112 |
| **7** | -0.172 | -0.169 | 0.120 | **-0.939** | -0.216 |
| 8 | 0.893 | -0.008 | 0.117 | -0.047 | 0.112 |
| 9 | 0.847 | 0.159 | 0.154 | 0.353 | 0.052 |
| 10 | 0.977 | -0.009 | 0.142 | -0.074 | -0.095 |
| 11 | 0.894 | 0.181 | 0.162 | 0.310 | 0.213 |
| 12 | 0.107 | 0.900 | -0.216 | 0.073 | 0.335 |
| **13** | 0.151 | **0.940** | 0.137 | 0.108 | 0.240 |
| **14** | -0.267 | 0.087 | **-0.921** | 0.133 | 0.000 |
| 15 | 0.258 | 0.184 | 0.787 | 0.004 | 0.188 |
| 16 | 0.113 | 0.931 | 0.133 | 0.047 | 0.282 |
| 17 | -0.128 | 0.227 | -0.909 | 0.177 | 0.146 |
| **18** | -0.149 | 0.383 | -0.132 | -0.117 | **0.666** |
| 19 | 0.042 | 0.590 | -0.182 | 0.188 | -0.171 |

1.Annual Mean Temperature; 2.Mean Diurnal Range (Mean of monthly (max temp - min temp)); 3.Isothermality; 4.Temperature Seasonality (standard deviation *100); 5.Max Temperature of Warmest Month; 6.Min Temperature of Coldest Month; 7.Temperature Annual Range; 8.Mean Temperature of Wettest Quarter; 9.Mean Temperature of Driest Quarter; 10.Mean Temperature of Warmest Quarter; 11.Mean Temperature of Coldest Quarter; 12.Annual Precipitation; 13.Precipitation of Wettest Month; 14.Precipitation of Driest Month; 15.Precipitation Seasonality (Coefficient of Variation); 16.Precipitation of Wettest Quarter; 17.Precipitation of Driest Quarter; 18.Precipitation of Warmest Quarter; 19.Precipitation of Coldest Quarter.

**Table S10.** Contemporary occurrence records (237) of *Tabebuia aurea* represented by the centroid of grid cells across the Neotropics used in the ecological niche modelling (ENM)**.**

| **Long** | **Lat** |  | **Long** | **Lat** |  | **Long** | **Lat** |  | **Long** | **Lat** |
| --- | --- | --- | --- | --- | --- | --- | --- | --- | --- | --- |
| -62.25 | -14.25 |  | -51.25 | -22.25 |  | -47.75 | -18.25 |  | -42.25 | -13.75 |
| -61.75 | -14.75 |  | -51.25 | -18.75 |  | -47.75 | -16.75 |  | -42.25 | -13.25 |
| -61.25 | -15.25 |  | -51.25 | -18.25 |  | -47.75 | -16.25 |  | -42.25 | -9.75 |
| -61.25 | -14.75 |  | -51.25 | -16.75 |  | -47.75 | -15.75 |  | -41.75 | -13.25 |
| -60.75 | -14.75 |  | -51.25 | -14.75 |  | -47.75 | -15.25 |  | -41.75 | -4.75 |
| -60.75 | -13.75 |  | -50.75 | -22.25 |  | -47.75 | -9.25 |  | -41.75 | -4.25 |
| -60.25 | -17.75 |  | -50.75 | -17.75 |  | -47.75 | -6.25 |  | -41.25 | -16.25 |
| -60.25 | -15.75 |  | -50.75 | -14.25 |  | -47.25 | -22.25 |  | -41.25 | -12.75 |
| -59.75 | -18.25 |  | -50.25 | -24.75 |  | -47.25 | -20.25 |  | -41.25 | -8.25 |
| -59.25 | -19.25 |  | -50.25 | -22.75 |  | -47.25 | -19.25 |  | -40.75 | -9.75 |
| -59.25 | -18.25 |  | -50.25 | -20.75 |  | -47.25 | -15.75 |  | -40.25 | -9.25 |
| -59.25 | -14.25 |  | -50.25 | -16.75 |  | -47.25 | -15.25 |  | -40.25 | -7.75 |
| -58.75 | -17.75 |  | -50.25 | -16.25 |  | -47.25 | -13.75 |  | -39.75 | -10.75 |
| -58.25 | -19.75 |  | -50.25 | -15.75 |  | -47.25 | -6.75 |  | -39.75 | -9.25 |
| -58.25 | -16.75 |  | -50.25 | -13.25 |  | -47.25 | -6.25 |  | -39.75 | -8.75 |
| -58.25 | -16.25 |  | -49.75 | -20.75 |  | -46.75 | -22.25 |  | -39.25 | -16.25 |
| -57.75 | -19.25 |  | -49.75 | -17.25 |  | -46.75 | -18.75 |  | -39.25 | -12.75 |
| -57.75 | -17.75 |  | -49.75 | -10.75 |  | -46.75 | -17.75 |  | -39.25 | -10.25 |
| -57.75 | -16.25 |  | -49.25 | -24.25 |  | -46.75 | -17.25 |  | -39.25 | -9.75 |
| -57.25 | -22.75 |  | -49.25 | -22.25 |  | -46.75 | -14.25 |  | -39.25 | -9.25 |
| -57.25 | -22.25 |  | -49.25 | -19.25 |  | -46.75 | -13.75 |  | -39.25 | -8.75 |
| -57.25 | -21.75 |  | -49.25 | -16.75 |  | -46.75 | -10.75 |  | -39.25 | -7.25 |
| -57.25 | -19.75 |  | -49.25 | -16.25 |  | -46.25 | -18.75 |  | -39.25 | -5.25 |
| -57.25 | -17.75 |  | -49.25 | -15.75 |  | -46.25 | -16.25 |  | -38.75 | -9.75 |
| -56.75 | -20.75 |  | -49.25 | -15.25 |  | -46.25 | -13.25 |  | -38.75 | -8.75 |
| -56.75 | -19.75 |  | -49.25 | -14.25 |  | -45.75 | -16.25 |  | -38.75 | -4.25 |
| -56.75 | -19.25 |  | -49.25 | -13.75 |  | -45.75 | -15.25 |  | -38.75 | -3.75 |
| -56.75 | -18.75 |  | -49.25 | -12.75 |  | -45.75 | -12.25 |  | -38.25 | -11.25 |
| -56.75 | -16.75 |  | -49.25 | -12.25 |  | -45.75 | -11.75 |  | -38.25 | -10.25 |
| -56.75 | -16.25 |  | -49.25 | -11.75 |  | -45.25 | -16.75 |  | -38.25 | -9.75 |
| -56.25 | -20.25 |  | -48.75 | -23.25 |  | -45.25 | -16.25 |  | -38.25 | -9.25 |
| -56.25 | -15.75 |  | -48.75 | -22.75 |  | -45.25 | -12.75 |  | -38.25 | -8.75 |
| -56.25 | -14.25 |  | -48.75 | -22.25 |  | -45.25 | -12.25 |  | -38.25 | -6.75 |
| -55.75 | -22.25 |  | -48.75 | -20.75 |  | -45.25 | -11.25 |  | -38.25 | -6.25 |
| -55.75 | -21.25 |  | -48.75 | -20.25 |  | -45.25 | -10.25 |  | -37.75 | -9.75 |
| -55.75 | -20.25 |  | -48.75 | -19.25 |  | -45.25 | -9.75 |  | -37.75 | -9.25 |
| -55.75 | -16.25 |  | -48.75 | -18.75 |  | -45.25 | -7.25 |  | -37.75 | -8.25 |
| -55.75 | -15.25 |  | -48.75 | -17.75 |  | -44.75 | -14.25 |  | -37.25 | -10.75 |
| -55.75 | -14.75 |  | -48.75 | -16.25 |  | -44.75 | -13.25 |  | -37.25 | -10.25 |
| -55.75 | -9.75 |  | -48.75 | -15.75 |  | -44.75 | -12.25 |  | -37.25 | -9.75 |
| -55.25 | -21.75 |  | -48.75 | -13.75 |  | -44.75 | -11.75 |  | -37.25 | -8.25 |
| -55.25 | -20.25 |  | -48.75 | -8.75 |  | -44.75 | -11.25 |  | -37.25 | -7.25 |
| -54.75 | -22.75 |  | -48.75 | -8.25 |  | -44.75 | -10.75 |  | -37.25 | -6.75 |
| -54.75 | -22.25 |  | -48.75 | -0.75 |  | -44.25 | -20.75 |  | -36.75 | -10.25 |
| -54.75 | -20.25 |  | -48.25 | -22.75 |  | -44.25 | -19.25 |  | -36.75 | -9.75 |
| -54.75 | -14.75 |  | -48.25 | -18.75 |  | -44.25 | -15.25 |  | -36.75 | -8.75 |
| -54.75 | -2.75 |  | -48.25 | -18.25 |  | -44.25 | -13.75 |  | -36.75 | -7.25 |
| -54.75 | -1.75 |  | -48.25 | -15.75 |  | -44.25 | -4.25 |  | -36.75 | -6.75 |
| -53.25 | -22.25 |  | -48.25 | -14.25 |  | -44.25 | -3.75 |  | -36.75 | -6.25 |
| -52.75 | -16.25 |  | -48.25 | -13.75 |  | -44.25 | -2.75 |  | -36.25 | -9.25 |
| -52.25 | -21.75 |  | -48.25 | -13.25 |  | -43.75 | -19.75 |  | -36.25 | -8.25 |
| -52.25 | -15.75 |  | -48.25 | -10.25 |  | -43.75 | -18.75 |  | -36.25 | -7.75 |
| -52.25 | -14.75 |  | -48.25 | -8.25 |  | -43.75 | -18.25 |  | -36.25 | -7.25 |
| -52.25 | -13.75 |  | -48.25 | -7.25 |  | -43.75 | -15.25 |  | -35.75 | -8.25 |
| -51.75 | -20.75 |  | -48.25 | -5.75 |  | -43.75 | -6.75 |  | -35.75 | -6.75 |
| -51.75 | -18.25 |  | -47.75 | -22.75 |  | -43.25 | -9.25 |  | -35.25 | -7.75 |
| -51.75 | -17.75 |  | -47.75 | -22.25 |  | -43.25 | -4.75 |  | -35.25 | -5.75 |
| -51.75 | -17.25 |  | -47.75 | -21.25 |  | -42.75 | -10.75 |  |  |  |
| -51.75 | -16.75 |  | -47.75 | -19.75 |  | -42.75 | -5.25 |  |  |  |
| -51.75 | -12.75 |  | -47.75 | -19.25 |  | -42.75 | -3.75 |  |  |  |

**Table S11**. Ecological niche modelling methods used to estimate *Tabebuia aurea* potential distribution.

| **Method** | **Species data type** |
| --- | --- |
| Bioclimatic Envelope (BIOCLIM) | Presence only |
| Ecological Niche Factor Analysis (ENFA) | Presence only |
| Euclidian Distance (EuclidDist) | Presence only |
| Generalized Linear Models (GLM) | Presence and absence |
| Gower Distance (GowerDist) | Presence only |
| Mahalanobis Distance (MahalDist) | Presence only |
| Maximum Entropy (Maxent) | Presence/background |
| Generalized additive models (GAM) | Presence and absence |
| Flexible discriminant analysis (FDA) | Presence and absence |
| Multivariate adaptive regression splines (MARS) | Presence and absence |
| Generalized boosted models (GBM) | Presence and absence |
| Neural Networks (ANN) | Presence and absence |
| Random Forest (RNDFOR) | Presence and absence |

**Table S12**. Values of True Skill Statistics (TSS), with mean and standard deviation, for all ENM*AOGCM’s combinations from ecological niche modelling of *Tabebuia aurea*.

|  |  | **AOGCMs** | | | | | | |
| --- | --- | --- | --- | --- | --- | --- | --- | --- |
|  |  | | CCSM | CNRM | MIROC | MRI | **Mean** | **SD** |
| **ENM Algorithms** | **BioClim** | | 0.5 | 0.6 | 0.5 | 0.5 | 0.5 | 0.1 |
|  | **ENFA** | | 0.4 | 0.4 | 0.4 | 0.3 | 0.4 | 0.0 |
|  | **EuclidDist** | | 0.5 | 0.5 | 0.4 | 0.5 | 0.5 | 0.1 |
|  | **FDA** | | 0.6 | 0.6 | 0.6 | 0.6 | 0.6 | 0.0 |
|  | **GAM** | | 0.7 | 0.7 | 0.6 | 0.6 | 0.6 | 0.0 |
|  | **GBM** | | 0.7 | 0.6 | 0.6 | 0.6 | 0.6 | 0.1 |
|  | **GLM** | | 0.6 | 0.6 | 0.6 | 0.6 | 0.6 | 0.0 |
|  | **GowerDist** | | 0.5 | 0.6 | 0.5 | 0.5 | 0.5 | 0.1 |
|  | **MahalanobisDist** | | 0.5 | 0.6 | 0.5 | 0.5 | 0.5 | 0.1 |
|  | **MARS** | | 0.6 | 0.6 | 0.5 | 0.6 | 0.6 | 0.0 |
|  | **MaxEnt** | | 0.6 | 0.6 | 0.6 | 0.6 | 0.6 | 0.0 |
|  | **NNet** | | 0.6 | 0.6 | 0.6 | 0.6 | 0.6 | 0.0 |
|  | **RndFor** | | 0.7 | 0.7 | 0.6 | 0.6 | 0.7 | 0.0 |
|  | **Mean** | | 0.6 | 0.6 | 0.5 | 0.5 | *0.6* | *0.0* |
|  | **SD** | | 0.1 | 0.1 | 0.1 | 0.1 | *0.1* |  |

**Table S13.** Sampling location for the 20 populations of *Tabebuia aurea* from Brazil, used for genetic analyses.

| Code | Population Locality | Latitude | Longitude |
| --- | --- | --- | --- |
| **AGE** | Águas Emendadas Ecological Reserve, DF | S15 35 04.7 | W47 39 59.7 |
| **ARA** | Formoso do Araguaia, TO | S11 33 43.9 | W49 48 15.9 |
| **BAG** | Barra do Garça, MT | S15 00 47.3 | W51 16 41.7 |
| **BAR** | Barreiras, BA | S12 05 06.3 | W45 26 50.8 |
| **BOD** | Aquidauana, MS | S20 35 59.2 | W56 00 12.4 |
| **CAC** | Cáceres, MT | S16 08 23.7 | W57 59 19.8 |
| **CHG** | Chapada dos Guimarães, MT | S15 23 45.0 | W55 50 00.7 |
| **FAT** | Fátima, TO | S10 48 20.8 | W48 54 22.9 |
| **GSV** | Chapada Gaúcha, MG | S15 13 25.6 | W45 49 12.0 |
| **NIQ** | Niquelândia, GO | S14 11 39.7 | W48 18 38.2 |
| **PAN** | Januária, MG | S15 30 53.6 | W44 41 32.5 |
| **PNE** | Mineiros, GO | S17 54 43.2 | W52 21 08.9 |
| **POT** | Portelândia, GO | S17 19 31.6 | W52 42 17.1 |
| **PTU** | Paracatu, MG | S17 27 44.3 | W46 39 26.5 |
| **SCA** | Serra da Canastra, MG | S19 34 25.6 | W46 29 56.1 |
| **SDO** | Serra Dourada, GO | S16 04 02.1 | W50 10 35.6 |
| **SEC** | Campo Maior, PI | S4 51 37.4 | W42 03 36.5 |
| **STZ** | Santa Terezinha de Goiás, GO | S14 21 07.0 | W49 30 15.8 |
| **SUM** | Lagoa Santa, MG | S19 32 24.3 | W43 56 02.2 |
| **VIB** | Vila Boa, GO | S15 00 46.0 | W47 02 32.9 |
