# Supplementary material for: Recovering species demographic history from multi-model inference: the case of a Neotropical savanna tree species
Source: BMC Evol Biol. 2014 Oct 11;14:213. doi: 10.1186/s12862-014-0213-0 (PMC4205293; doi:10.1186/s12862-014-0213-0)
Supplement: Additional file 2: Figures S1-S7. — Additional data o ecological niche modelling, population structure analyses and spatial pattern in genetic diversity. [file 12862_2014_213_MOESM2_ESM.doc]

**Additional file 2**

**
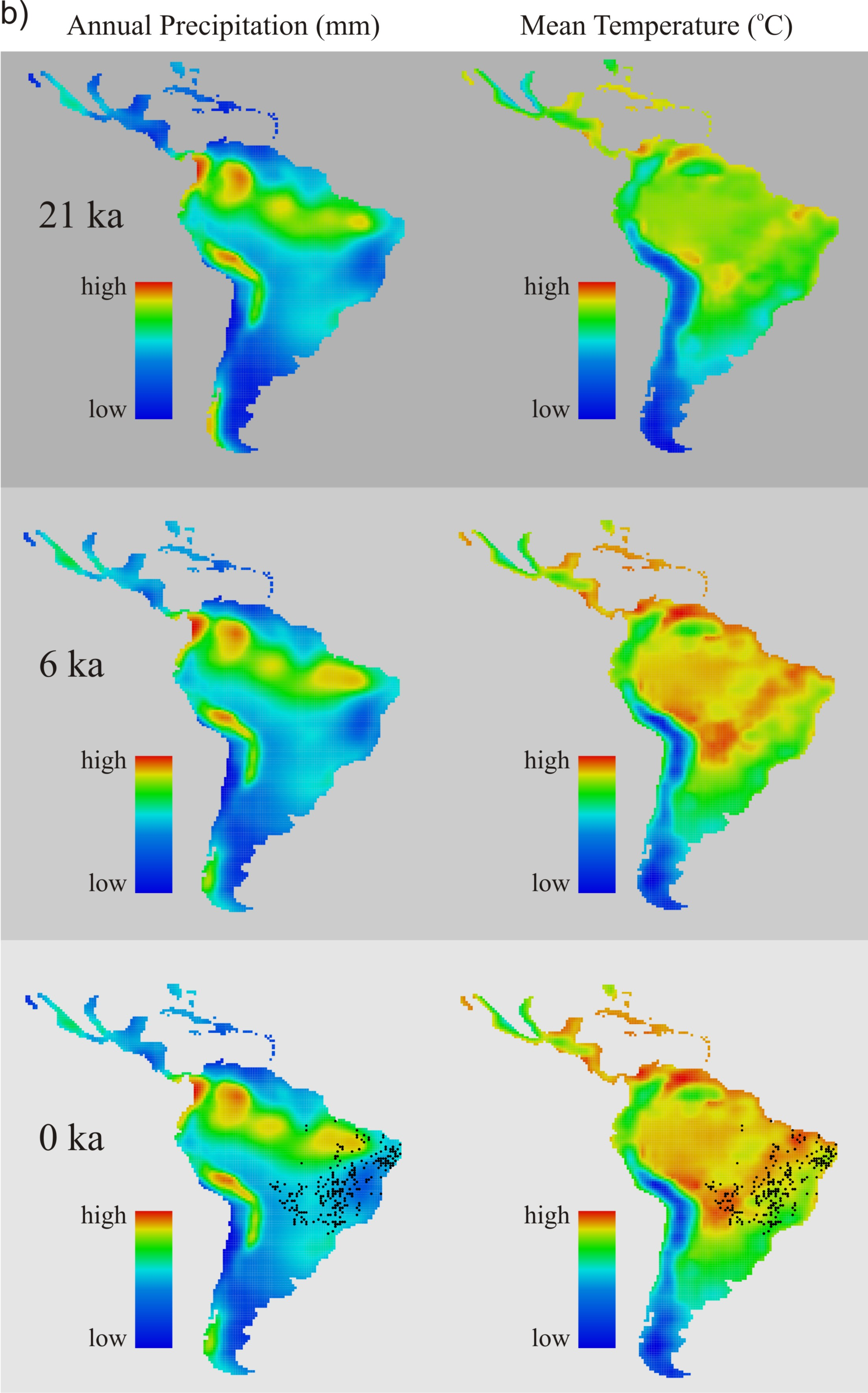
**

**Figure S1.** Geographical space of climatic variables in Neotropics during the LGM (21 ka), mid-Holocene (6 ka) and present-day (0 ka). The bioclimatic variables were obtained from AOGCM CCSM4.


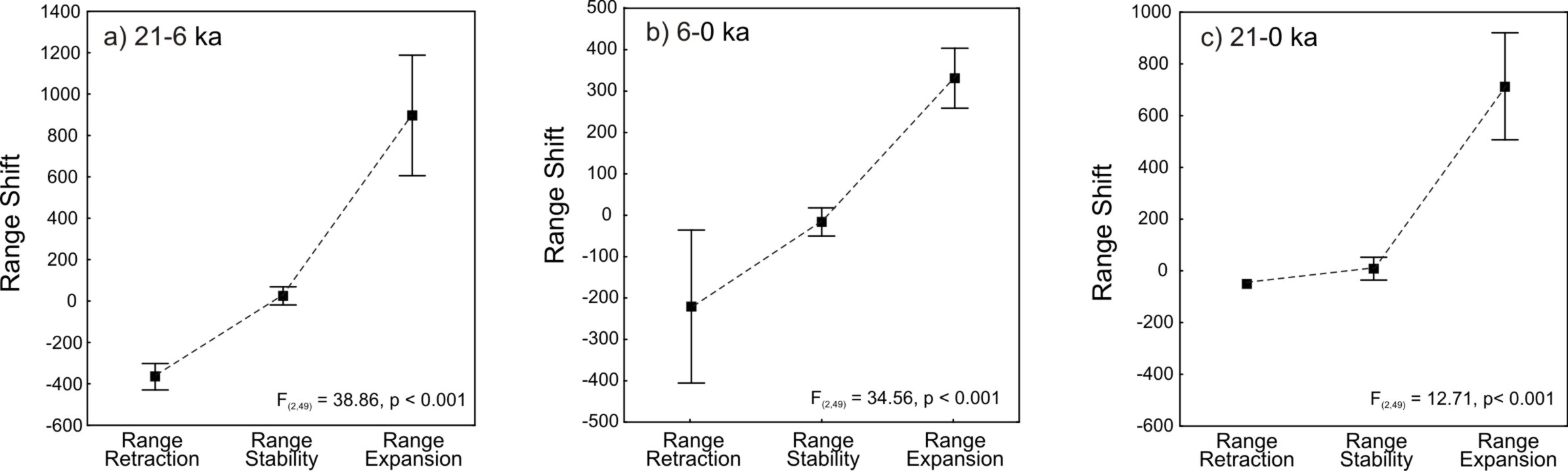


**Figure S2.** Average and 0.95 confidence interval of range shift predicted by palaeodistribution modelling for *Tabebuia aurea* in each scenario and time slice: (a) 21kyr -6kyr - range size at 6 ka minus 21 ka; (b) 6kyr - 0kyr - range size at 0 ka minus 6 ka; and (c) 21kyr - 0kyr - range size at 0 ka minus 21 ka. In (c), the range retraction does not present confidence interval because just two maps predicted that scenario between LGM and present. Note that the average of range shift predicted by 52 maps follows systematically the expected by each general scenario (positive range shifts for range expansion, negative range shifts for range retraction, and no variation for range stability).


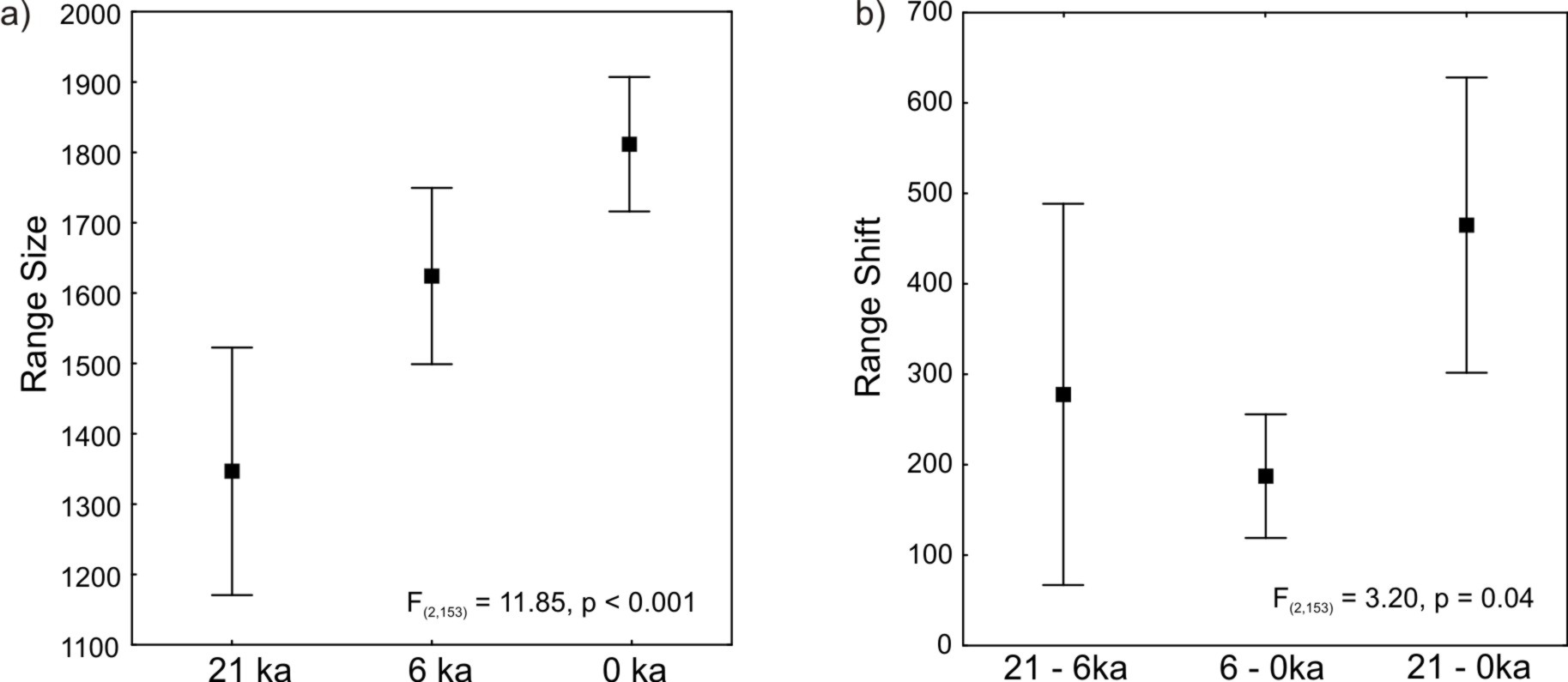


**Figure S3.** Average and 0.95 confidence interval among the 52 maps of (a) range size and (b) shift (difference of range size among time periods) predicted for *Tabebuia aurea* at LGM (21 ka), mid-Holocene (6 ka), and present-day (0 ka). Note that a general scenario of range expansion though time (positive range shifts) is supported by maps classification.

**Figure S4.** Posterior probability graphs of the Bayesian clustering simulation implemented in the software STRUCTURE 2.3.3 (Pritchard et al. 2000) for 414 individuals of *Tabebuia aurea* in Central Brazil.


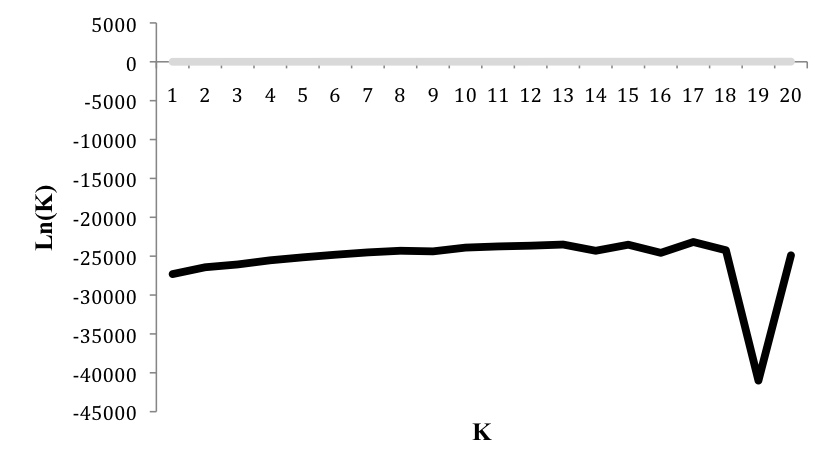


**Figure S4a**. Log of the posterior probability of data [L(D|K)] as a function of K averaged over 5 independent runs for *Tabebuia aurea*, derived using a Bayesian clustering algorithm implemented in the software STRUCTURE.


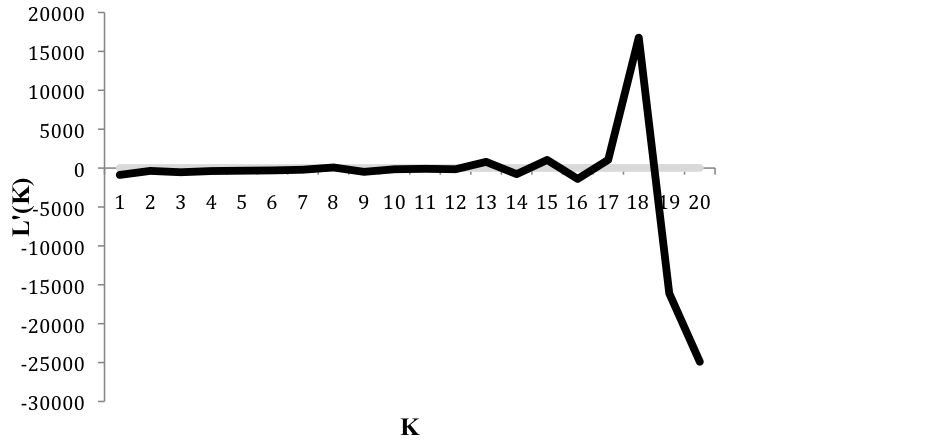


**Figure S4b**. Differences between the log of the posterior probability of the data {[L(D|K)_n_ - [L(D|K)_n-1_ } as a function of K averaged over 5 independent runs for *Tabebuia aurea*, derived using a Bayesian clustering algorithm implemented in the software STRUCTURE.


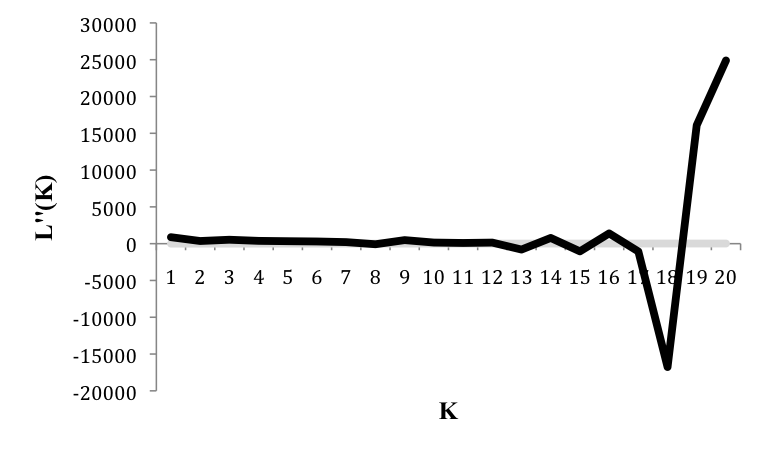


**Figure S4c.** Module of the differences between the log of the posterior probability of the data |{[L(D|K)_n_ as- [L(D|K)_n-1_ }| as a function of K averaged over 5 independent runs for *Tabebuia aurea*, derived using a Bayesian clustering algorithm implemented in the software STRUCTURE.


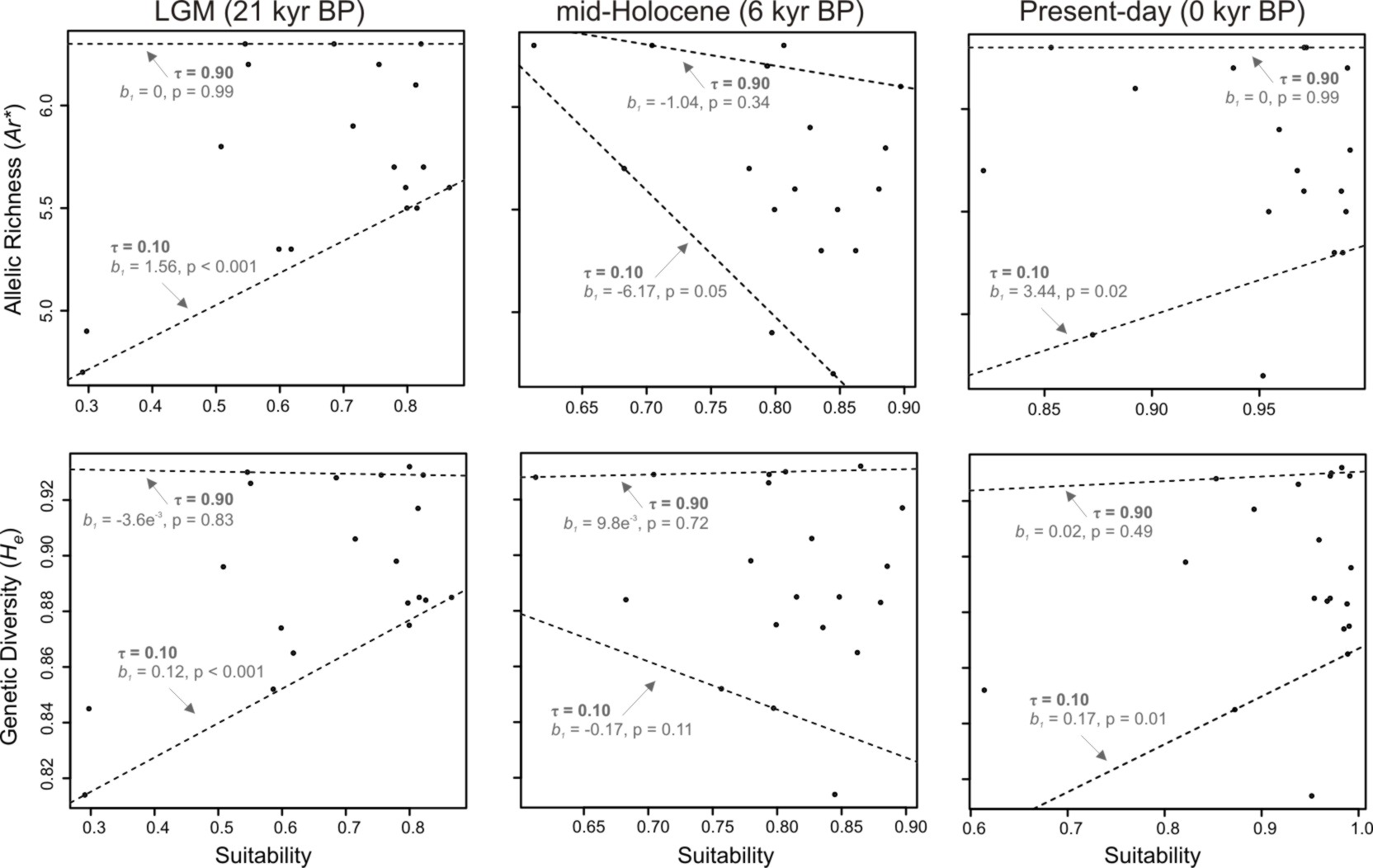


**Figure S5.** Quantile regression for allelic richness (*Ar**) and genetic diversity (*He*) with climatic suitability for 20 populations of *Tabebuia aurea* at the LGM (21 kyr BP), mid-Holocene (6 kyr BP), and present-day (0 kyr BP). Dashed lines show the fitness for 90% (upper) and 10% (down) quantiles.


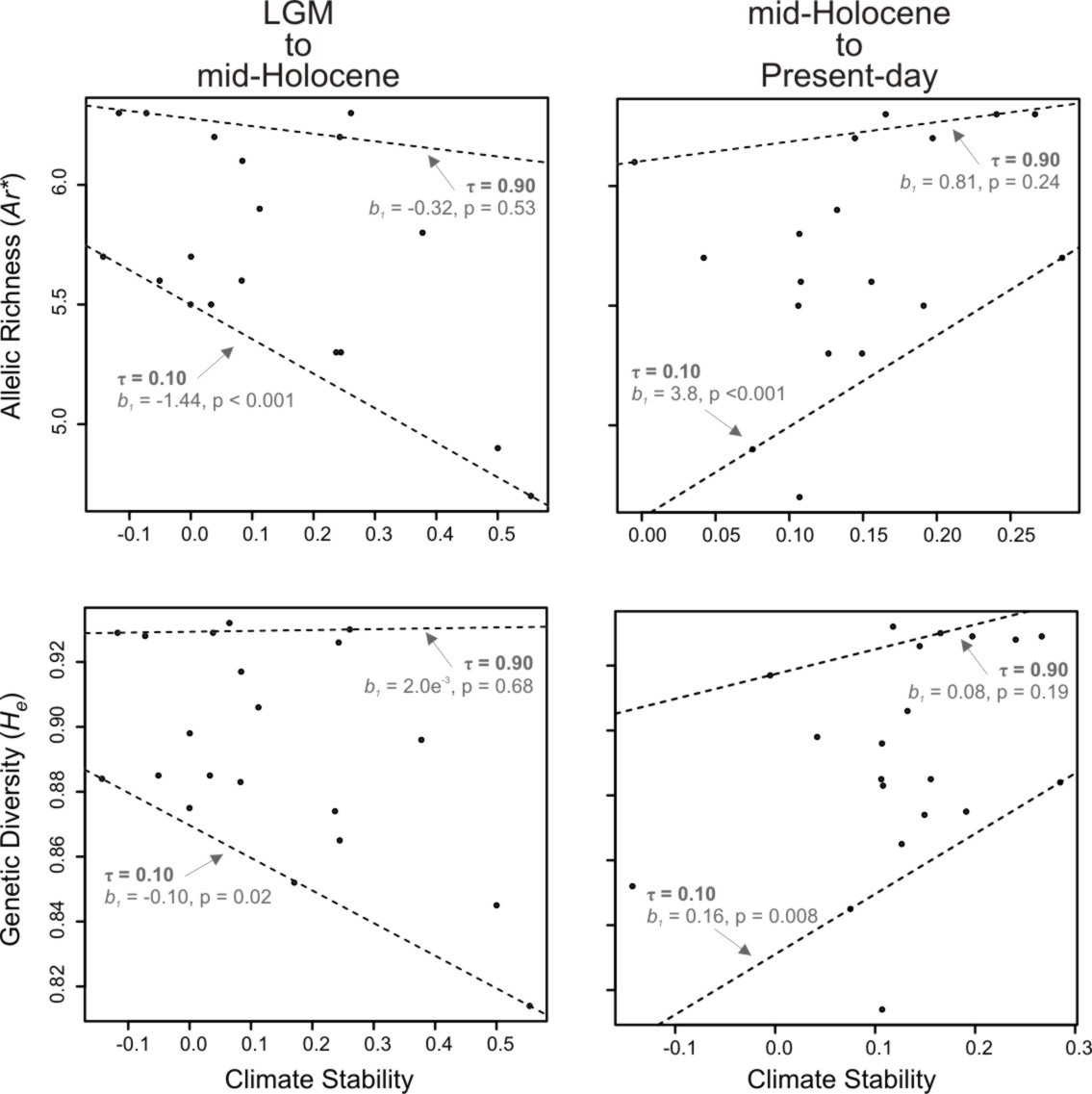


**Figure S6.** Quantile regression for both allelic richness (*Ar**) and genetic diversity (*He*) with climate stability for 20 populations of *Tabebuia aurea* from LGM to mid-Holocene and from mid-Holocene to present-day. Dashed lines show the fitness for 90% (upper) and 10% (down) quantiles.


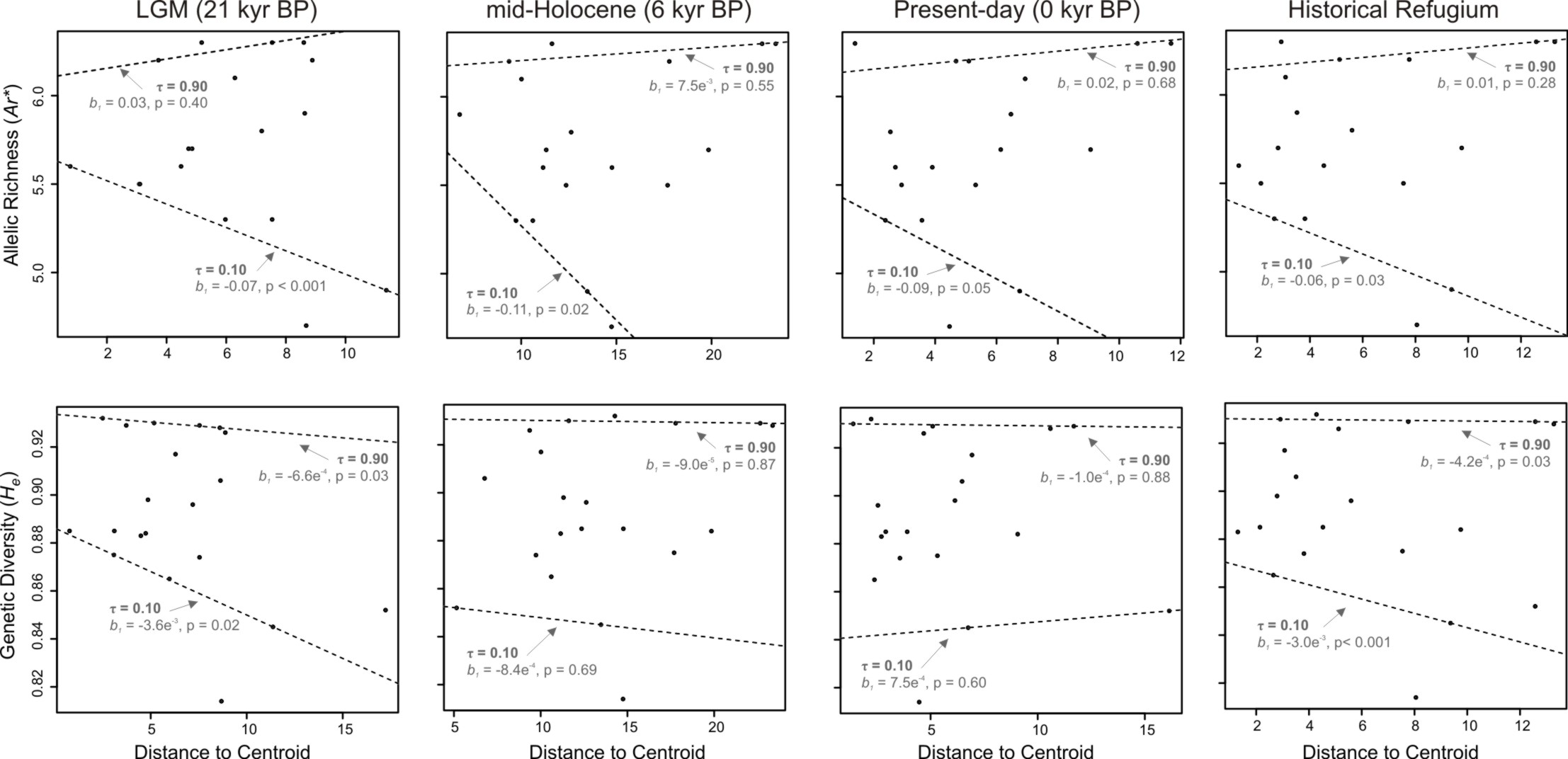


**Figure S7.** Quantile regression for allelic richness (*Ar**) and genetic diversity (*He*) with distance from the centroid of *Tabebuia aurea's* geographical range at present-day (0 kyr BP), mid-Holocene (6 kyr BP), and LGM (21 kyr BP), and distance from the centroid of the historical refugium. Dashed lines show the fitness for 90% (upper) and 10% (down) quantiles.
