# Supplementary material for: Recovering species demographic history from multi-model inference: the case of a Neotropical savanna tree species
Source: BMC Evol Biol. 2014 Oct 11;14:213. doi: 10.1186/s12862-014-0213-0 (PMC4205293; doi:10.1186/s12862-014-0213-0)
Supplement: Additional file 3: — Details on genotyping procedure. [file 12862_2014_213_MOESM3_ESM.doc]

**Additional file 3**

**Details on genotyping procedure**

To genotype all individuals (414), we used 11 nuclear microsatellite loci previously developed for *T. aurea* [1]. Primers were marked with fluorescent dyes (6-FAM, HEX and NED, Applied Biosystems, CA). Microsatellite loci amplifications were performed for each locus separately, in a 15μL volume containing 0.26 μM of each primer, 1U Taq DNA polymerase (Phoneutria, BR), 250 mM of each dNTP, 1X reaction buffer (10 mM Tris-HCl, pH 8.3, 50 mM KCl, 1.5 mM MgCL_2_), 300 mg of BSA and 9.0 ng of template DNA. Amplifications were performed using a PE9700 thermal controller (Applied Biosystems, CA) under the following conditions: 94°C for 5 min (one cycle); 94°C for 1 min, 56°C for 1 min (annealing temperature), 72°C for 1 min (35 cycles); and 72°C for 30 min (one cycle).

Fragments were subjected to electrophoresis on an ABI Prism 3100 automated DNA sequencer (Applied Biosystems, CA) and were sized by comparison to a 500 internal lane standard ROX (Applied Biosystems, CA). Fluorescent PCR products were automatically sized using GeneMaper v4.1 software (Applied Biosystems, CA).

Micro-Checker software [2] was used to detect errors due to stutter bands, allele dropout and null alleles. The analysis of raw data showed no significant evidence of genotyping errors or null alleles.

**Additional References**:

1. Braga A, Reis A, Leoi L, Pereira R, Collevatti R. 2007 Development and characterization of microsatellite markers for the tropical tree species *Tabebuia aurea* (Bignoniaceae). *Molecular Ecology Notes* **7**, 53-56.

2. van Oosterhout C, Hutchinson W, Wills D, Shipley P. 2004 MICRO-CHECKER: software for identifying and correcting genotyping errors in microsatellite data. *Molecular Ecology Notes* **4**, 535-538.
